# Cross-Species Reactivity and Differential Anti-PRRSV Activities of CD163 SRCR4/SRCR5 Monoclonal Antibodies

**DOI:** 10.3390/vetsci13080835

**Published:** 2026-08-20

**Authors:** Yifan Meng, Shuai Yang, Jun Jiao, Pingping Zhang, Qinchao Guan, Yanhan Lin, Meng Cui, Xinkai Wang, Xiaoyang Zhu, Ming Qiu, Hong Lin, Wanglong Zheng, Jianzhong Zhu, Ana M. M. Stoian, Lorenzo Fraile, Nanhua Chen

**Affiliations:** 1 College of Veterinary Medicine, Yangzhou University, Yangzhou 225009, China; m1653385177@163.com (Y.M.); ys15238113570@163.com (S.Y.); dz120230031@stu.yzu.edu.cn (J.J.); ydzp0229@163.com (P.Z.); 17853406351@163.com (Q.G.); 17757186305@163.com (Y.L.); cuimeng201902@163.com (M.C.); 15014175180@163.com (X.W.); zhuxiaoyang1998@126.com (X.Z.); qiuming1997@126.com (M.Q.); linhonglynn@yzu.edu.cn (H.L.); wanglongzheng@yzu.edu.cn (W.Z.); jzzhu@yzu.edu.cn (J.Z.); 2 Department of Animal Science (ETSEA), University of Lleida, 25198 Lleida, Spain; ana.stoian@udl.cat (A.M.M.S.); lorenzo.fraile@udl.cat (L.F.); 3 Jiangsu Co-Innovation Center for Prevention and Control of Important Animal Infectious Diseases and Zoonoses, Yangzhou University, Yangzhou 225009, China; 4 Comparative Medicine Research Institute, Yangzhou University, Yangzhou 225009, China

**Keywords:** CD163, monoclonal antibodies (mAbs), epitopes, cross-species reactivity, anti-PRRSV activities

## Abstract

CD163 not only acts as an essential receptor for porcine reproductive and respiratory syndrome virus (PRRSV) infection but also serves as a barrier to cross-species cellular infection by arteriviruses. Among its domains, the scavenger receptor cysteine-rich 5 (SRCR5) domain of CD163 is functionally indispensable. In this study, two monoclonal antibodies (mAbs), designated 5A and 11D, were developed against the SRCR4-6 region of porcine CD163. The linear epitopes recognized by mAbs 5A and 11D were identified as ^546^CEGHESHLSLCPVAP^560^ in SRCR5 and ^449^WDCKNW^454^ in SRCR4, respectively. Crystal structural analysis and sequence alignment showed that the ^546^CEGHESHLSLCPVAP^560^ epitope resides in the long loop 5-6 of SRCR5, which is a key structural region responsible for ligand binding. Meanwhile, the ^449^WDCKNW^454^ epitope is located within a highly conserved region of SRCR4, which confers broad cross-species reactivity. Notably, mAb 5A reduced PRRSV infection, while mAb 11D exhibited no anti-PRRSV activity in target cells. Collectively, this study provides essential molecular tools for exploring CD163-domain-dependent cross-species reactivity and developing anti-PRRSV strategies.

## Simple Summary

Porcine reproductive and respiratory syndrome virus (PRRSV) infection causes substantial economic losses to the global swine industry. PRRSV invades host target cells via receptor-mediated endocytosis, and CD163 is the most important receptor for PRRSV entry. Among the CD163 domains, the scavenger receptor cysteine-rich 5 (SRCR5) domain is essential for PRRSV infection. Therefore, CD163 and its SRCR5 are commonly selected as targets for developing anti-PRRSV strategies, including for the generation of CD163-SRCR5 knockout pigs. In addition, CD163 divergence also serves as a barrier to cross-species cellular infection by arteriviruses. Nevertheless, CD163 monoclonal antibodies (mAbs) with defined cross-species reactivity and anti-PRRSV activities have rarely been characterized. Here, we developed two mAbs against porcine CD163. Epitope mapping demonstrated that mAb 5A recognizes a novel epitope within the long loop 5-6 of the SRCR5 domain, while mAb 11D targets a conserved epitope within the SRCR4 domain. Functional validation revealed that mAb 5A can inhibit PRRSV infection, while mAb 11D cannot. Remarkably, mAb 11D exhibits broader cross-species reactivity than mAb 5A. Overall, this study provides a solid foundation for elucidating CD163-domain-dependent biological functions.

## 1. Introduction

The CD163 gene contains 17 exons, which encode a signal peptide, nine tandem scavenger receptor cysteine-rich (SRCR) domains (SRCR1 to SRCR9), two proline serine threonine (PST) linker peptides, a transmembrane domain and a small intracellular cytoplasmic tail [1]. The CD163 protein belongs to the class B SRCR superfamily and is mainly expressed on monocytes and macrophages [2]. The main physiological function of CD163 is the clearance of cell-free hemoglobin (Hb) and Hb–haptoglobin (Hb-Hp) complexes from the blood, thereby protecting the host from oxidative stress [2,3]. In addition, CD163 also participates in pro- and anti-inflammatory processes [1,4]. Soluble CD163 is a dynamic biomarker of liver inflammation in patients with bacterial infection and human immunodeficiency virus (HIV)/hepatitis C virus (HCV) coinfection [5,6]. Moreover, CD163 serves as a critical receptor for arterivirus infection, including simian hemorrhagic fever virus (SHFV) and porcine reproductive and respiratory syndrome virus (PRRSV) [7,8].

PRRSV infection causes reproductive failure in sows and respiratory diseases in piglets, resulting in substantial economic losses to the global swine industry [9]. PRRSV is an enveloped, positive-sense, single-stranded RNA virus belonging to the genus *Betaarterivirus*, family *Arteriviridae*, order *Nidovirales* [9,10]. The PRRSV genome is about 15 kb in length, encoding at least 16 nonstructural proteins (nsp1α, nsp1β, nsp2–nsp6, nsp2N, nsp2TF, nsp7α, nsp7β, and nsp8–nsp12) and eight structural proteins (GP2a, E, GP3, GP4, GP5, GP5a, M, and N) [11,12]. PRRSV enters susceptible cells via receptor-mediated endocytosis, and CD169 and CD163 were initially considered the major receptors mediating PRRSV entry into porcine alveolar macrophages (PAMs) [11,13,14]. The major envelope proteins GP5 and M interact with CD169 to mediate PRRSV internalization, while the minor glycoproteins GP2a and GP4 interact with CD163 to facilitate viral uncoating [13,14]. However, the generation of CD169-knockout pigs confirmed that CD169 is not required for PRRSV infection [15]. In contrast, CD163-knockout pigs are completely resistant to PRRSV infection, demonstrating that CD163 is essential for viral entry [16]. Further studies identified SRCR5 and PSTII as the key functional regions of CD163 required for PRRSV infection [17,18,19]. More precisely, the arginine residue at position 561 in SRCR5 has a significant impact on PRRSV replication [20]. On 29 April 2025, the U.S. Food and Drug Administration (FDA) approved CD163-SRCR5-knockout pigs for human consumption [21,22]. Nowadays, CD163-SRCR5 is a major target for the development of anti-PRRSV strategies.

Notably, CD163 varies considerably among species, creating a barrier to cross-species transmission of arteriviruses such as SHFV [8]. CD163 from apes is fully functional for SHFV entry, while CD163 from Old World monkeys and New World monkeys has significantly lower efficiency in supporting SHFV infection. Interestingly, human CD163 is fully functional for both SHFV and PRRSV infection [8,23]. Several monoclonal antibodies (mAbs) and nanobodies targeting porcine CD163 have been developed [24,25,26], but their cross-species reactivity has rarely been evaluated.

To provide essential molecular tools for studying CD163-domain-dependent cross-species reactivity and anti-PRRSV strategies, we generated two mAbs targeting the SRCR4-SRCR6 region of porcine CD163. Their epitopes were precisely identified, and their cross-species reactivity was evaluated using human, monkey, porcine and canine cells. Their anti-PRRSV activities were also assessed in target cells (PAMs and Marc-145). Collectively, this study provides a comprehensive foundation for exploring CD163-domain-dependent cross-species reactivity and anti-PRRSV strategies.

## 2. Materials and Methods

### 2.1. Cell Lines, Viruses, and Animals

The cell lines used in this study, including SP2/0 cells (Wuhan, China, Procell System Co., Ltd.), Marc-145 [11], HEK293T [27], 293T-pCD163 (HEK293T cells stably expressing porcine CD163) [28], SU-DHL-1 (ATCC: CRL-2955, Shanghai, China, WHELAB Co., Ltd.) and DH82 cells (Shanghai, China, Fuheng Biology Co., Ltd.), were maintained in Dulbecco’s Modified Eagle Medium (DMEM; HyClone, Logan, UT, USA) supplemented with 10% fetal bovine serum (FBS) (Inner Mongolia Jin Yuan Kang Biotechnology Co., Ltd., Hohhot City, China) and 1% penicillin–streptomycin (Solarbio, Beijing, China) at 37 °C in a humidified incubator with 5% CO_2_. PAMs were collected from bronchoalveolar lavage fluid of 6-week-old PRRSV-free piglets, as previously described [12,29], and cultured in RPMI 1640 medium (HyClone, Logan, UT, USA) supplemented with 10% FBS (EallBio, Beijing, China). The PRRSV strains used in this study were stored in our laboratory, including lineage 8 strains (HP-PRRSV-2 XJ17-5 and classical PRRSV-2 CH-1R) and lineage 1 strains (NADC34-like rBJ-VVL-P30 and NADC30-like rSD-VVL) [11,30,31]. Six-week-old specific-pathogen-free (SPF) female BALB/c mice were obtained from the Laboratory Animal Center of Yangzhou University [27]. All animal experiments were performed in accordance with the Guide for the Care and Use of Laboratory Animals (SYXK (JS) 2022-0044) and were authorized by the Institutional Animal Care and Use Committee of Yangzhou University.

### 2.2. RNA Extraction, cDNA Synthesis, and Plasmid Construction

Total RNA was extracted from PAMs using TRIpure reagent (Aidlab, Beijing, China) according to the manufacturer’s instructions [11]. The extracted RNA was then reverse transcribed into cDNA using reverse transcription reagents purchased from Vazyme Biotech Co., Ltd. (Nanjing, China) [28]. PCR amplification of the target fragment pCD163-SRCR4-6 was performed using the synthesized cDNA as a template with the primers pET32a-N-6his-F (No. 1) and pET32a-N-6his-R (No. 2) (Table S1). The PCR reaction was carried out using Phanta Flash polymerase (Vazyme, Nanjing, China). The resulting fragment was inserted into the prokaryotic expression vector pET32a-6His between the *BamHI* and *XhoI* cutting sites using the ClonExpress Ultra One Step Cloning Kit (Vazyme, Nanjing, China), generating the recombinant plasmid for expression of 6His-tagged pET32a-pCD163-SRCR4-6. The restriction endonucleases *BamHI* and *XhoI* were purchased from New England Biolabs (Ipswich, MA, USA). The fidelity of the obtained recombinant plasmid was confirmed by Sanger sequencing.

### 2.3. Expression and Purification of Recombinant Porcine CD163 Protein

The recombinant plasmid pET32a-pCD163-SRCR4-6 was transformed into competent *E. coli* BL21 (DE3) cells (TOLOBIO, Hefei, Anhui, China), and protein expression was induced with 1 mM isopropyl β-D-1-thiogalactopyranoside (IPTG) (Solarbio, Beijing, China) [27]. The recombinant protein was predominantly expressed in inclusion bodies, which were collected and solubilized in a buffer containing 8 M urea, followed by stepwise refolding through sequential dialysis against buffers with decreasing concentrations of urea (6 M, 4 M, 2 M, and 1 M) and a final dialysis step in phosphate-buffered saline (PBS) [32]. The expression and purification of pCD163-SRCR4-6 protein were assessed by SDS-PAGE followed by Coomassie brilliant blue staining (Beyotime Biotechnology, Shanghai, China). The identity of the recombinant protein was further confirmed by Western blotting using an anti-His tag monoclonal antibody (1:1000 dilution; TransGen Biotech, Beijing, China), as previously described [27,32].

### 2.4. Mouse Immunization and Preparation of Porcine CD163-Specific Monoclonal Antibodies

Porcine CD163-specific monoclonal antibodies were prepared as previously described [27,32]. Briefly, six-week-old female BALB/c mice were immunized subcutaneously with 200 μg of purified pCD163-SRCR4-6 protein emulsified in Montanide ISA adjuvant (SEPPIC, Paris, France). Two weeks after primary immunization, a booster dose of 100 μg of the same antigen was administered via the subcutaneous route, followed by two additional booster injections at one-week intervals. One week after the final booster, each mouse received an intraperitoneal injection of 100 μg purified pCD163-SRCR4-6 protein as a final boost. At 2–4 days post-injection, the immunized mice were euthanized, and splenocytes were aseptically harvested and fused with SP2/0 myeloma cells using PEG1500 (Boster Biological Technology, Wuhan, China) as the fusogen. After fusion, hybridoma clones secreting pCD163-SRCR4-6-specific antibodies were initially screened by indirect ELISA using the purified pCD163-SRCR4-6 protein as the coating antigen. Positive clones were subjected to three consecutive rounds of limiting dilution subcloning to establish stable monoclonal hybridoma cell lines. The specificity of the established monoclonal antibodies was further verified by Western blotting and indirect immunofluorescence assay (IFA) [12,29].

### 2.5. Antibody Purification

Monoclonal antibodies were purified from ascites fluid using Protein A/G PLUS-Agarose (Santa Cruz Biotechnology, sc-2003, Santa Cruz, CA, USA) [32]. Briefly, 500 μL of ascites fluid was mixed with 0.2 mL of Protein A/G agarose resin (50% slurry) in a 2 mL microcentrifuge tube and incubated at room temperature with end-over-end mixing for 10 min. The mixture was then centrifuged at 5000× *g* for 1 min, and the supernatant containing unbound components was carefully removed. The resin was washed three times with 400 μL of Binding Buffer (0.1 M phosphate, 0.15 M sodium chloride, pH 7.2), with centrifugation at 5000× *g* for 1 min between each wash. Bound antibodies were eluted by adding 400 μL of Elution Buffer (0.1 M glycine, pH 2.5) and incubating at room temperature for 5 min with gentle mixing, followed by centrifugation. The eluate was immediately neutralized with 40 μL of Neutralization Buffer (1 M Tris, pH 8.5). This elution step was repeated three times to obtain three eluted fractions. The absorbance of each fraction was measured at 280 nm to identify those containing purified antibodies. For downstream applications, the eluted antibodies were subjected to buffer exchange using Zeba Desalt Spin Columns (Thermo Scientific, Waltham, MA, USA) according to the manufacturer’s protocol. The resin was regenerated by washing with Elution Buffer and Storage Solution (0.02% sodium azide in PBS) and stored at 4 °C for future use.

### 2.6. pCD163 Protein-Coated Indirect ELISA

Indirect ELISA was performed to screen hybridoma clones secreting porcine CD163-specific antibodies [27]. Briefly, 96-well flat-bottom plates were coated overnight at 4 °C with 0.625 μg/mL recombinant pCD163-SRCR4-6 protein in carbonate–bicarbonate coating buffer (100 μL/well). The plates were then blocked with 5% skim milk in PBS at 37 °C for 2 h, followed by three washes with PBST (PBS containing 0.05% Tween-20). Hybridoma culture supernatants (100 μL/well) were added and incubated at 37 °C for 1 h. After washing, horseradish peroxidase (HRP)-conjugated goat anti-mouse IgG (1:10,000 dilution; TransGen Biotech, Beijing, China) was added (100 μL/well) and incubated at 37 °C for 1 h. The enzymatic reaction was developed by adding 3,3′,5,5′-tetramethylbenzidine (TMB) substrate (50 μL/well; Beyotime Biotech, Shanghai, China) and terminated with 2 M H_2_SO_4_ (50 μL/well). The optical density at 450 nm (OD_450_) was measured using a microplate reader (ALL-SHENG, Hangzhou, China). The positive-to-negative (P/N) ratio was calculated by comparing the OD_450_ values of hybridoma supernatants with those of the SP2/0 negative control supernatants, and clones with a P/N ratio of ≥2.1 were considered positive.

### 2.7. Construction of pCD163 Truncation Constructs for Epitope Identification

To identify the epitopes recognized by monoclonal antibodies (mAbs), a series of truncated pCD163-SRCR4-6 constructs was generated using the eukaryotic expression vector pEGFP-C1 [32]. The truncated sequences were cloned into the pEGFP-C1 vector between the *KpnI* and *BglII* restriction sites. Using the previously constructed plasmid pET32a-6His-pCD163-SRCR4-6 as a template, the fragments corresponding to pCD163-SRCR4, pCD163-SRCR5, pCD163-SRCR6, and pCD163-SRCR4-6 were amplified by PCR with the primer pairs No. 3/No. 4, No. 5/No. 6, No. 7/No. 8, and No. 3/No. 8, respectively (Table S1). These fragments were then cloned into the pEGFP-C1 vector between the *KpnI* and *BglII* cutting sites to generate plasmids expressing the pCD163 truncation mutants (372–472) AA, (477–577) AA, (582–682) AA, and (372–682) AA, respectively. The resulting constructs were transfected into 293T cells for expression of the corresponding EGFP-tagged fusion proteins. To further define the antibody epitopes, additional plasmids encoding EGFP-fused truncated CD163 derivatives were constructed by seamless cloning using specific PCR primers (Table S1).

### 2.8. Western Blotting (WB)

WB was carried out as previously described [12]. Briefly, cells were harvested and lysed in RIPA (radio-immunoprecipitation assay) buffer supplemented with protease and phosphatase inhibitors (CWBIO, Beijing, China). Equal amounts of proteins were separated by 8% SDS-PAGE under reducing conditions and electrotransferred onto polyvinylidene difluoride (PVDF) membranes (Cytiva, formerly GE Healthcare Life Sciences, Beijing, China). The membranes were blocked with 5% non-fat skim milk in Tris-buffered saline containing 0.1% Tween-20 (TBST) for 1 h at room temperature, followed by overnight incubation at 4 °C with primary antibodies at a 1:1000 dilution. After three washes with TBST, the blots were incubated with an HRP-conjugated goat anti-mouse secondary antibody (1:10,000; TransGen Biotech, Beijing, China) for 1 h at room temperature. Immunoreactive signals were detected using an ECL substrate (Tanon, Shanghai, China) and visualized with a chemiluminescence imaging system (Tanon, Shanghai, China).

### 2.9. Immunofluorescence Assay (IFA)

Cells were cultured in 24-well plates prior to immunofluorescence staining [28,29]. Briefly, cells were fixed with 4% paraformaldehyde (Beyotime Biotech, Shanghai, China) for 15 min at room temperature, followed by permeabilization with 0.1% Triton X-100 (Beyotime Biotechnology, Shanghai, China) in PBS for 15 min. The cells were then blocked with 2% bovine serum albumin (BSA) (Solarbio, Beijing, China) for 1 h to prevent nonspecific binding and incubated with porcine CD163-specific monoclonal antibodies (1:1000 dilution) at 4 °C overnight. After extensive washing, the cells were incubated with DyLight™ 594-conjugated goat anti-mouse IgG (H + L) secondary antibody (1:1000; Thermo Fisher Scientific, Sunnyvale, CA, USA) for 1 h at room temperature in the dark. Nuclei were stained with 4′,6-diamidino-2-phenylindole (DAPI; Thermo Fisher Scientific, Waltham, CA, USA) for 5 min. Fluorescent images were acquired using a Leica SPE confocal fluorescence microscope (Buffalo Grove, IL, USA).

### 2.10. Detection of Antiviral Effects

Marc-145 cells were cultured in 24-well plates and pre-incubated with monoclonal antibodies at concentrations of 10 μg/mL and 5 μg/mL in DMEM for 1 h at 37 °C. Subsequently, the cells were infected with the PRRSV strains XJ17-5, rBJ-VVL-P30, rSD-VVL and CH-1R at an MOI of 0.1. At 48 h post-infection, the cells were harvested for Western blotting. To validate the antiviral activity of the monoclonal antibodies in primary target cells, PAMs were treated under similar conditions but with different antibody concentrations (50 μg/mL and 100 μg/mL), and PRRSV infection at 24 hpi was evaluated by IFA [32].

## 3. Results

### 3.1. pCD163-SRCR4-6 Cloning, Expression and Purification

**Figure 1:**
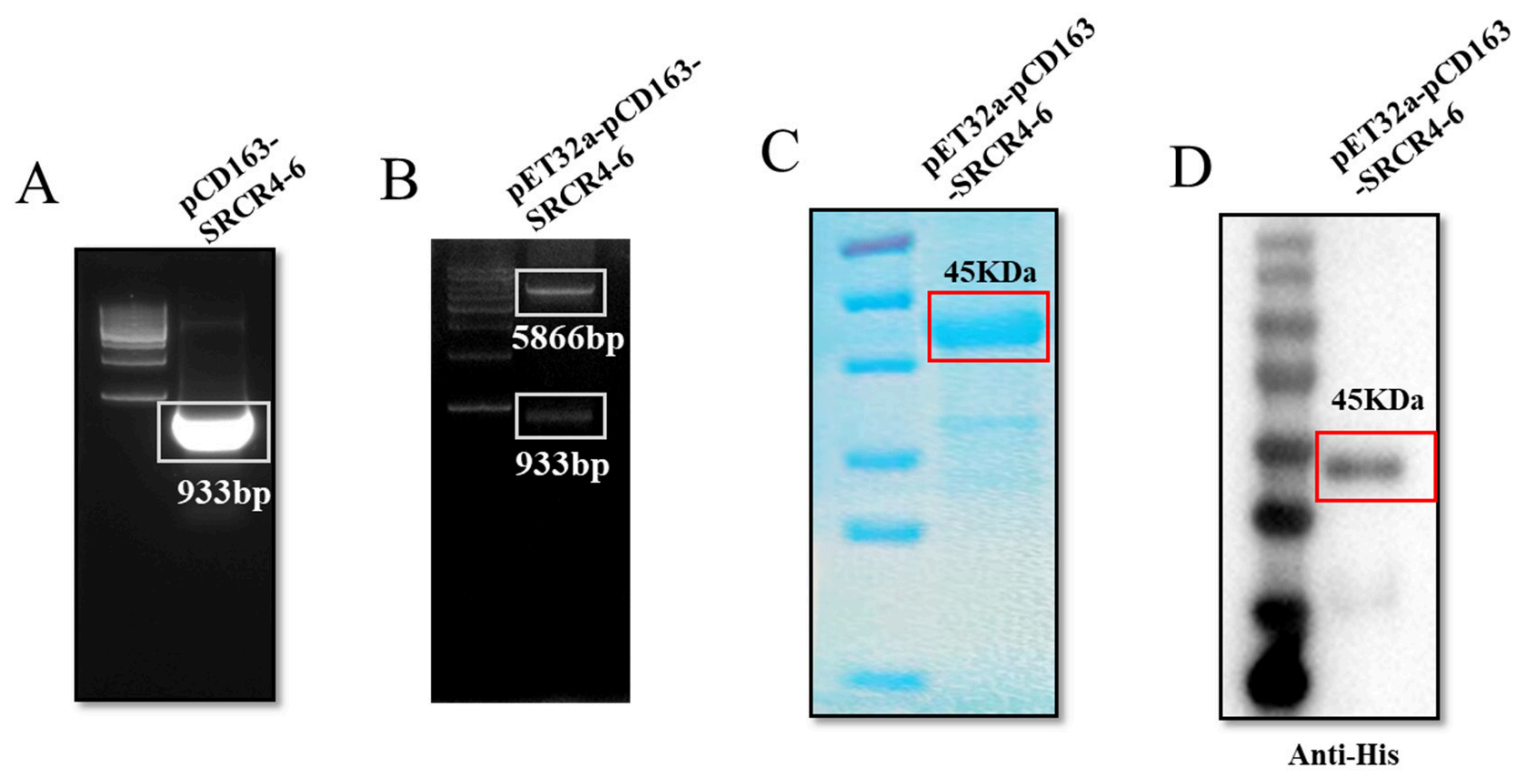
The expression and purification of pET32a-pCD163-SRCR4-6. (**A**) PCR amplification of the pCD163-SRCR4-6 fragment. (**B**) Double restriction enzyme digestion of the prokaryotic expression plasmid pET32a-pCD163-SRCR4-6 by *BamHI* and *XhoI*. (**C**,**D**) The recombinant plasmid was transformed into competent *E. coli* BL21 cells. Protein expression was induced with 1 mmol/L IPTG at 37 °C for 6 h. The expression of the fusion protein pET32a-pCD163-SRCR4-6 was confirmed by Coomassie brilliant blue staining and Western blotting.

The pCD163-SRCR4-6 fragment, with a size of 933 bp, was successfully amplified from PAMs (Figure 1A). Subsequently, the amplicon was inserted into the prokaryotic expression vector pET32a-6His to generate a pET32a-pCD163-SRCR4-6 recombinant plasmid, which was confirmed by restriction enzyme digestion with *BamHI* and *XhoI* (Figure 1B). The recombined pET32a-pCD163-SRCR4-6 protein was expressed in *E. coli* BL21 (DE3) cells, predominantly as inclusion bodies (Figure 1C). The pET32a-pCD163-SRCR4-6 protein was then purified via urea gradient centrifugation, which was verified by Western blotting using an anti-His mAb (Figure 1D).

### 3.2. Development and Characterization of Monoclonal Antibodies Against pCD163-SRCR4-6

**Figure 2:**
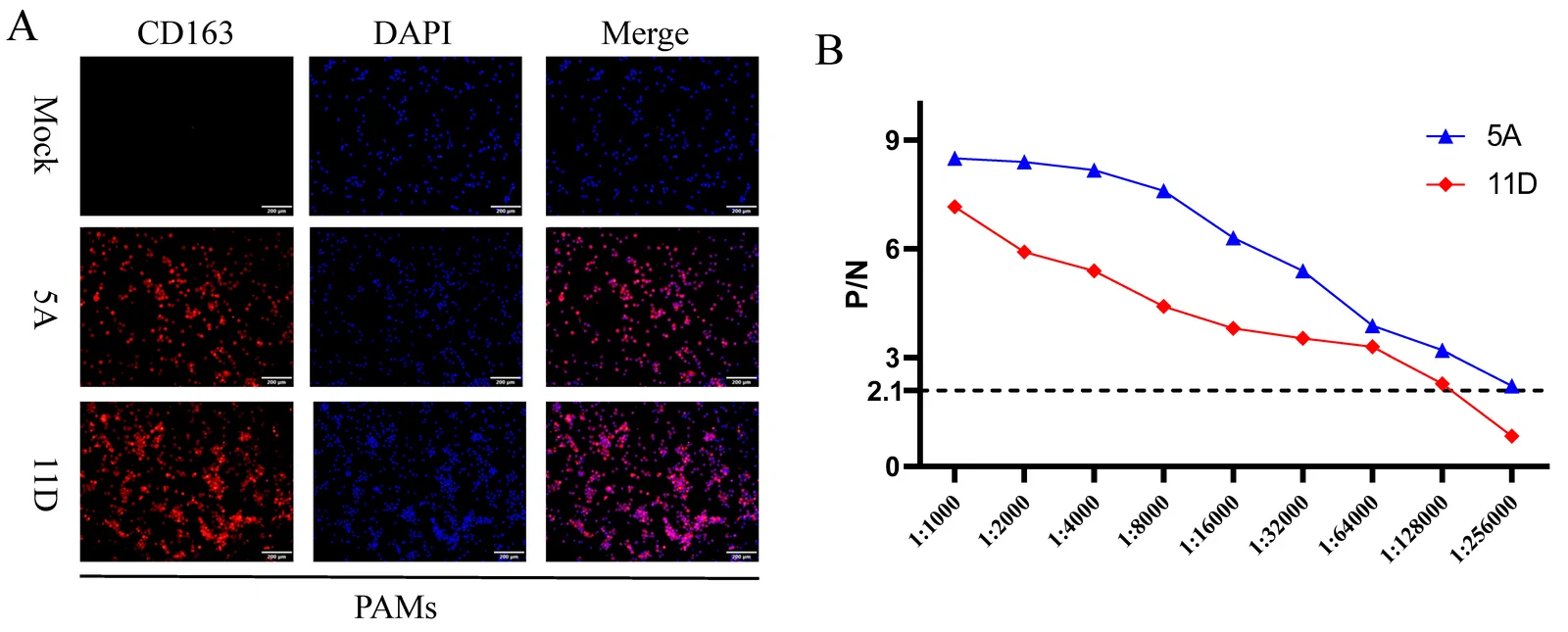
The generation of mAbs against pCD163-SRCR4-6. (**A**) IFA was performed to detect native CD163 expression in PAMs at 24 h after seeding by mAbs 5A and 11D. (**B**) The antibody titers of ascites-type antibodies containing mAbs 5A and 11D were determined by pCD163 protein-coated indirect ELISA. The antibody titer of each sample was determined as the highest dilution factor at which the P/N ratio exceeded 2.1.

Two porcine CD163 mAbs (5A and 11D) were obtained through indirect ELISA screening and three rounds of subcloning via limiting dilution. The specificity of both mAbs was confirmed by IFA, which demonstrated specific detection of endogenous CD163 in PAMs (Figure 2A). The antibody titers of ascites-type antibodies (endpoint titer defined as P/N ≥ 2.1) were determined by pCD163 protein-coated indirect ELISA as 1:256,000 for mAb 5A and 1:128,000 for mAb 11D (Figure 2B). Collectively, these results demonstrated the successful generation of two mAbs against pCD163.

### 3.3. Precise Epitope Mapping of mAbs 5A and 11D

**Figure 3:**
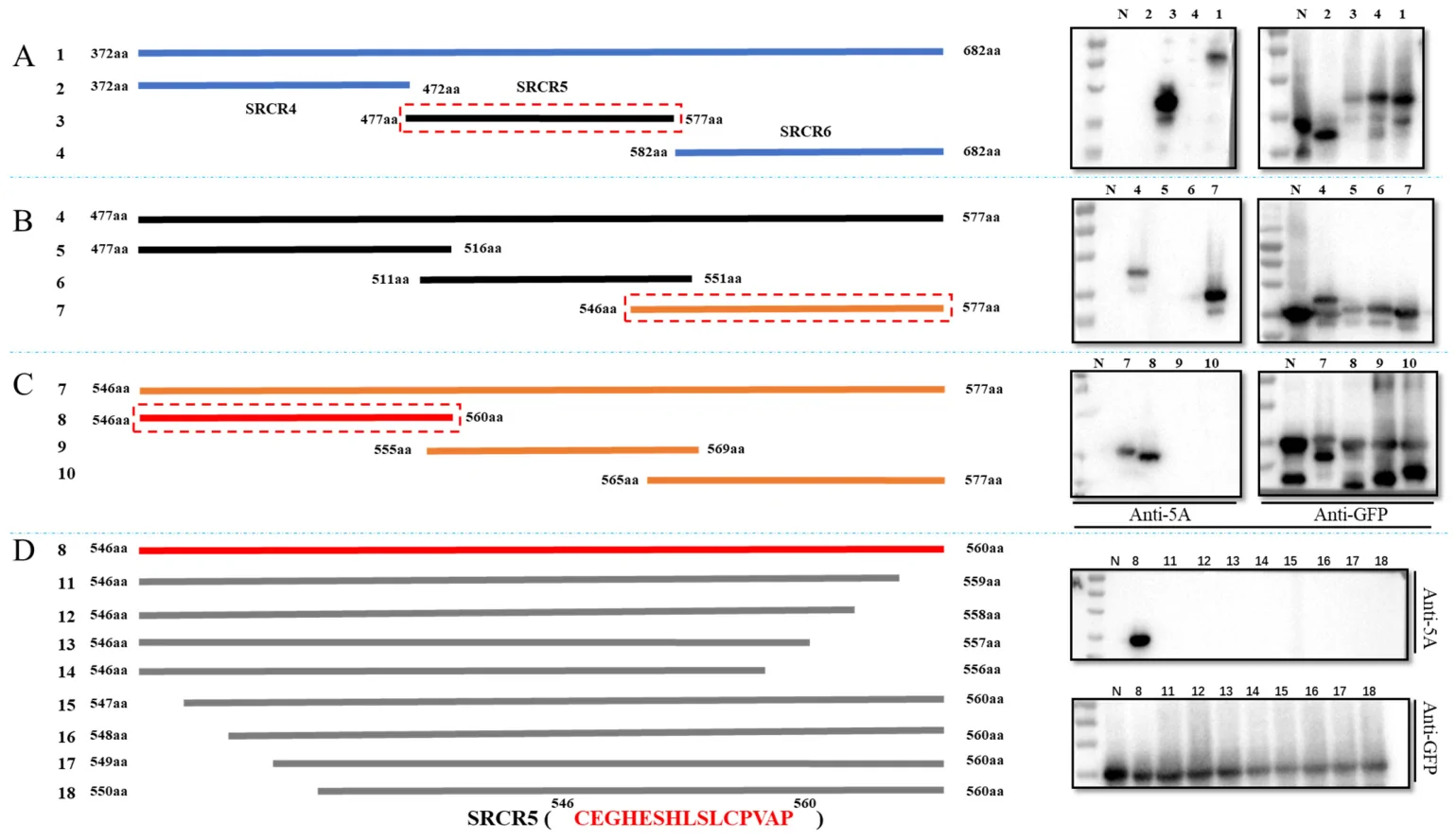
Epitope mapping of mAb 5A. Figures (**A**) through (**D**) are directly separated by blue dashed lines. (**A**) (**Left**): Constructions of pCD163-SRCR4-6 ((372–682) AA) and three truncations ((372–472) AA, (477–577) AA, (582–682) AA). (**Right**): WB results indicate the truncation recognized by mAb 5A. (**B**) The construction of truncations based on (477–577) AA (**Left**) and corresponding WB results (**Right**). (**C**) The construction of truncations based on (546–577) AA (**Left**) and corresponding WB results (**Right**). (**D**) The construction of truncations based on (546–560) AA (**Left**) and corresponding WB results (**Right**). The numbers 1–18 correspond to pCD163-SRCR4-6 and 17 truncated mutants. The red dashed boxes indicate truncations positively reacting with mAb 5A. The “N” in the WB results indicates the empty pEGFP-C1 vector.

Western blotting using full-length pCD163-SRCR4-6 (amino acids 372–682) and three truncated constructs encompassing (372–472) AA, (477–577) AA, and (582–682) AA revealed that the epitope recognized by mAb 5A was located within the SRCR5 domain (amino acids 477–577) (Figure 3A). To further refine the epitope, additional truncation mutants were constructed using the primer pairs No. 3/No. 9, No. 10/No. 11, and No. 12/No. 4, which generated constructs expressing the pCD163 truncations (477–516) AA, (511–551) AA, and (546–577) AA, respectively. The corresponding fusion proteins were obtained, and Western blot analysis narrowed the epitope region to (546–577) AA (Figure 3B). Subsequently, a further series of truncations was generated using the primer pairs No. 12/No. 13, No. 14/No. 15, and No. 16/No. 17 in the same manner, producing the truncated proteins (546–560) AA, (555–569) AA, and (565–577) AA. This step confined the epitope to the (546–560) AA region (Figure 3C). Finally, sequential truncation of each amino acid within the (546–560) AA region was performed using primer pair Nos. 20–37 to generate the following truncation mutants: (546–559) AA, (546–558) AA, (546–557) AA, (546–556) AA, (547–560) AA, (548–560) AA, (549–560) AA, and (550–560) AA. Western blot analysis identified the minimal epitope recognized by mAb 5A as the linear peptide sequence “CEGHESHLSLCPVAP”, corresponding to amino acids 546–560 of the SRCR5 domain (Figure 3D).

**Figure 4:**
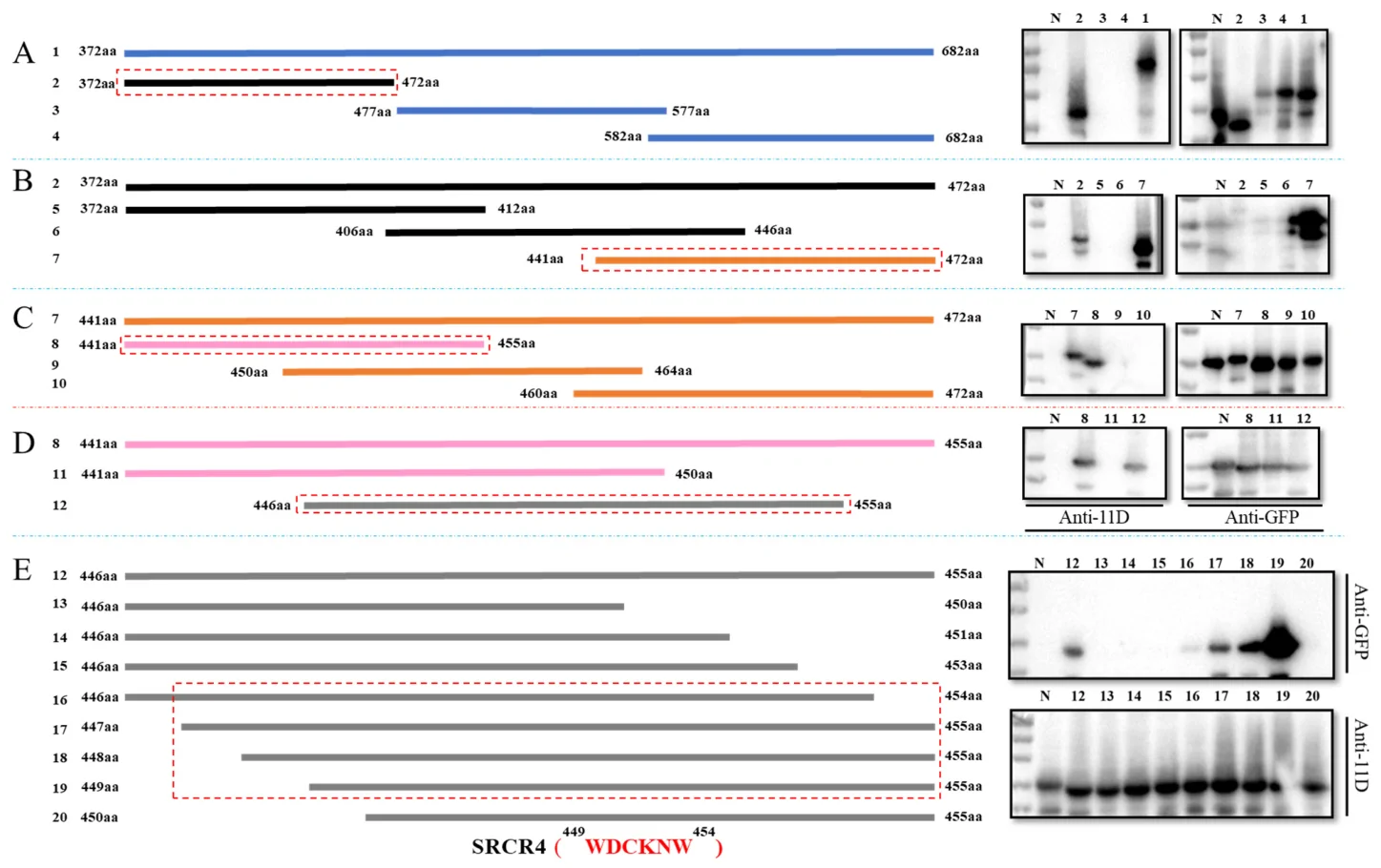
Epitope mapping of mAb 11D. Figures (**A**) through (**E**) are directly separated by blue dashed lines. (**A**) (**Left**): Constructions of pCD163-SRCR4-6 ((372–682) AA) and three truncations ((372–472) AA, (477–577) AA, (582–682) AA). (**Right**): WB results indicate the truncation recognized by mAb 11D. (**B**) The construction of truncations based on (372–472) AA (**Left**) and corresponding WB results (**Right**). (**C**) The construction of truncations based on (441–472) AA (**Left**) and corresponding WB results (**Right**). (**D**) The construction of truncations based on (441–455) AA (**Left**) and corresponding WB results (**Right**). (**E**) The construction of truncations based on (446–455) AA (**Left**) and corresponding WB results (**Right**). The numbers 1–20 correspond to pCD163-SRCR4-6 and 19 truncated mutants. The red dashed boxes indicate the truncations’ positive reaction with mAb 11D. The “N” in the WB results indicates pEGFP-C1 empty vector.

Using the same approach, the epitope recognized by mAb 11D was initially mapped to the (372–472) AA region of SRCR4 by Western blot analysis of the pCD163 truncation mutants (372–472) AA, (477–577) AA, (582–682) AA, and (372–682) AA (Figure 4A). Subsequently, a similar stepwise truncation strategy was employed using primer pair Nos. 38–71 to generate a series of progressively truncated SRCR4 constructs. Progressive epitope mapping by Western blot analysis ultimately identified the minimal epitope recognized by mAb 11D as the linear peptide sequence “WDCKNW,” corresponding to amino acid residues 449–454 of the SRCR4 domain (Figure 4B–E).

**Figure 5:**
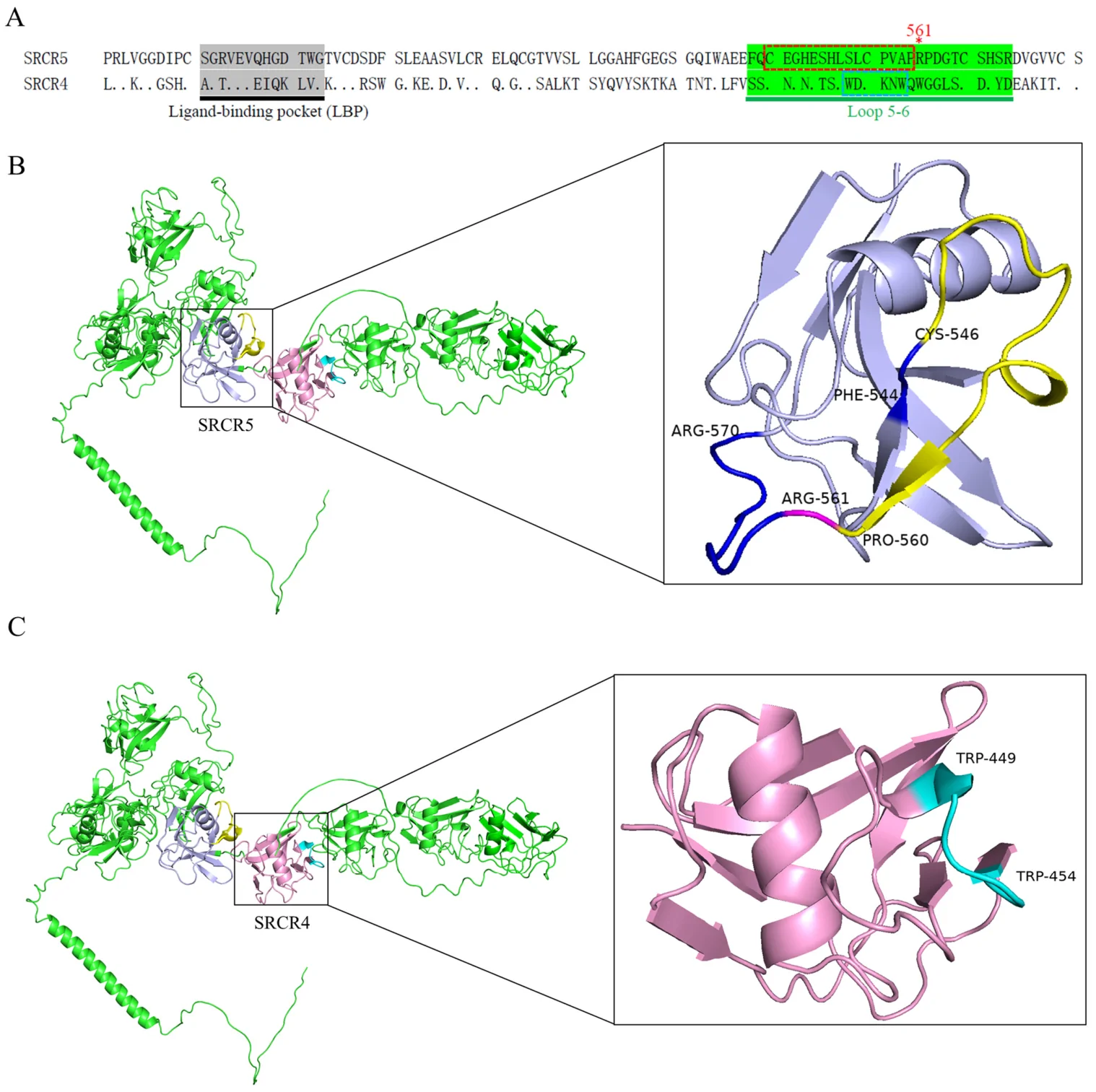
Epitope locations in the aligned sequences of SRCR4/SRCR5 and the crystal structure of pCD163. (**A**) Sequence alignment of SRCR4 and SRCR5. The ligand-binding pocket (LBP) and long loop 5-6 region are highlighted. The epitopes recognized by 5A and 11D are shown in different color boxes. The arginine residue at position 561 (R561) is marked with a red asterisk (*). (**B**) The crystal structure of pCD163 and SRCR5. The long loop 5-6 (544–570) is shown in dark blue. The ^546^CEGHESHLSLCPVAP^560^ epitope is marked in yellow. R561 is highlighted in fuchsia. (**C**) The crystal structure of pCD163 and SRCR4. The ^449^WDCKNW^454^ epitope is shown in turquoise. The AlphaFold-predicted crystal structure of pCD163 (UniProt ID: Q2VL90 · C163A_PIG) was obtained from the UniProt database and rendered with PyMOL 3.1.8.

To facilitate visualization of the epitopes recognized by the two mAbs, we mapped their locations onto the sequence alignment of SRCR4/SRCR5 and the AlphaFold-predicted crystal structure of pCD163 (Figure 5). Notably, the epitopes ^546^CEGHESHLSLCPVAP^560^ and ^449^WDCKNW^454^ recognized by mAbs 5A and 11D, respectively, are both located within the long loop 5-6 regions of SRCR5 and SRCR4 (Figure 5A). The long loop 5-6 of CD163 has been proposed as a potential ligand-binding region [33,34]. Moreover, the ^546^CEGHESHLSLCPVAP^560^ epitope is located near the R561 residue in SRCR5, which has been shown to play a critical role in PRRSV infection [20]. These results suggest that the two mAbs may interfere with PRRSV entry by targeting functionally important regions in CD163.

### 3.4. Anti-PRRSV Effects of mAbs 5A and 11D

**Figure 6:**
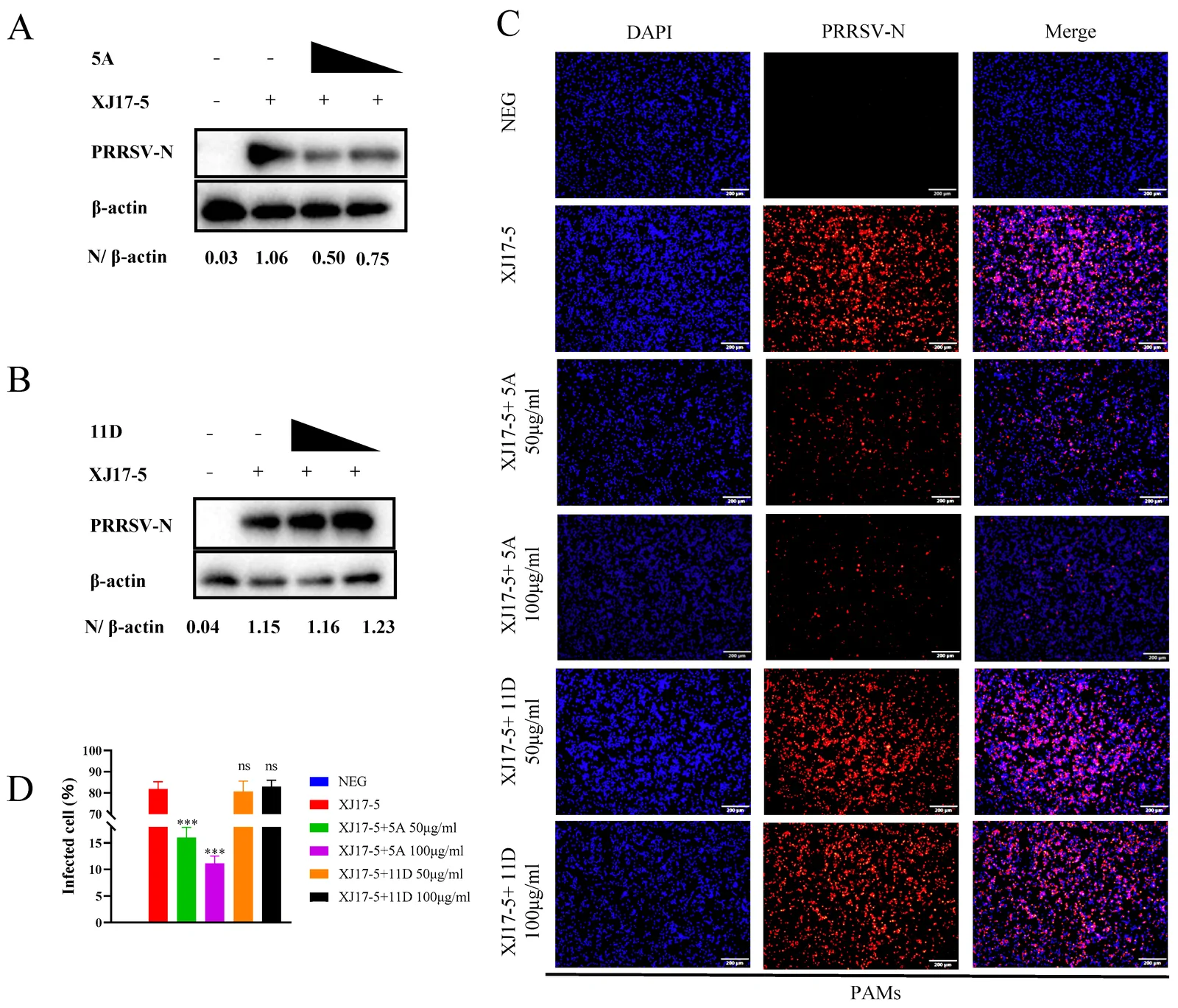
Anti-PRRSV effects of mAbs 5A and 11D in Marc-145 cells and PAMs. (**A**,**B**) mAb 5A, but not mAb 11D, dose-dependently decreased XJ17-5 infection in Marc-145 cells at 48 hpi. (**C**,**D**) The antiviral activity against XJ17-5 in PAMs at 24 hpi. The statistical analyses were performed by comparing to XJ17-5. *** indicated significant difference (*p* < 0.001), while ns indicated no significant difference.

To determine the anti-PRRSV activities of mAbs 5A and 11D, we evaluated their effects on infection by the HP-PRRSV-2 strain XJ17-5 in Marc-145 cells and PAMs (Figure 6). Western blotting showed that mAb 5A reduced XJ17-5 infection in Marc-145 cells in a dose-dependent manner at 48 hpi (Figure 6A), while mAb 11D had no detectable effect on XJ17-5 infection (Figure 6B). Consistent with these findings, IFA confirmed the dose-dependent anti-PRRSV activity of mAb 5A, whereas mAb 11D did not inhibit PRRSV infection in PAMs (Figure 6C,D). To evaluate the breadth of antiviral activities, additional PRRSV strains were tested in Marc-145 cells. MAb 5A also dose-dependently inhibited infection by the NADC34-like strain rBJ-VVL-P30 but showed no antiviral activity against the NADC30-like strain rSD-VVL or the classical PRRSV strain CH-1R (Figure S1).

### 3.5. Cross-Species Reactivity of Two mAbs Against pCD163-SRCR4-6

**Figure 7:**
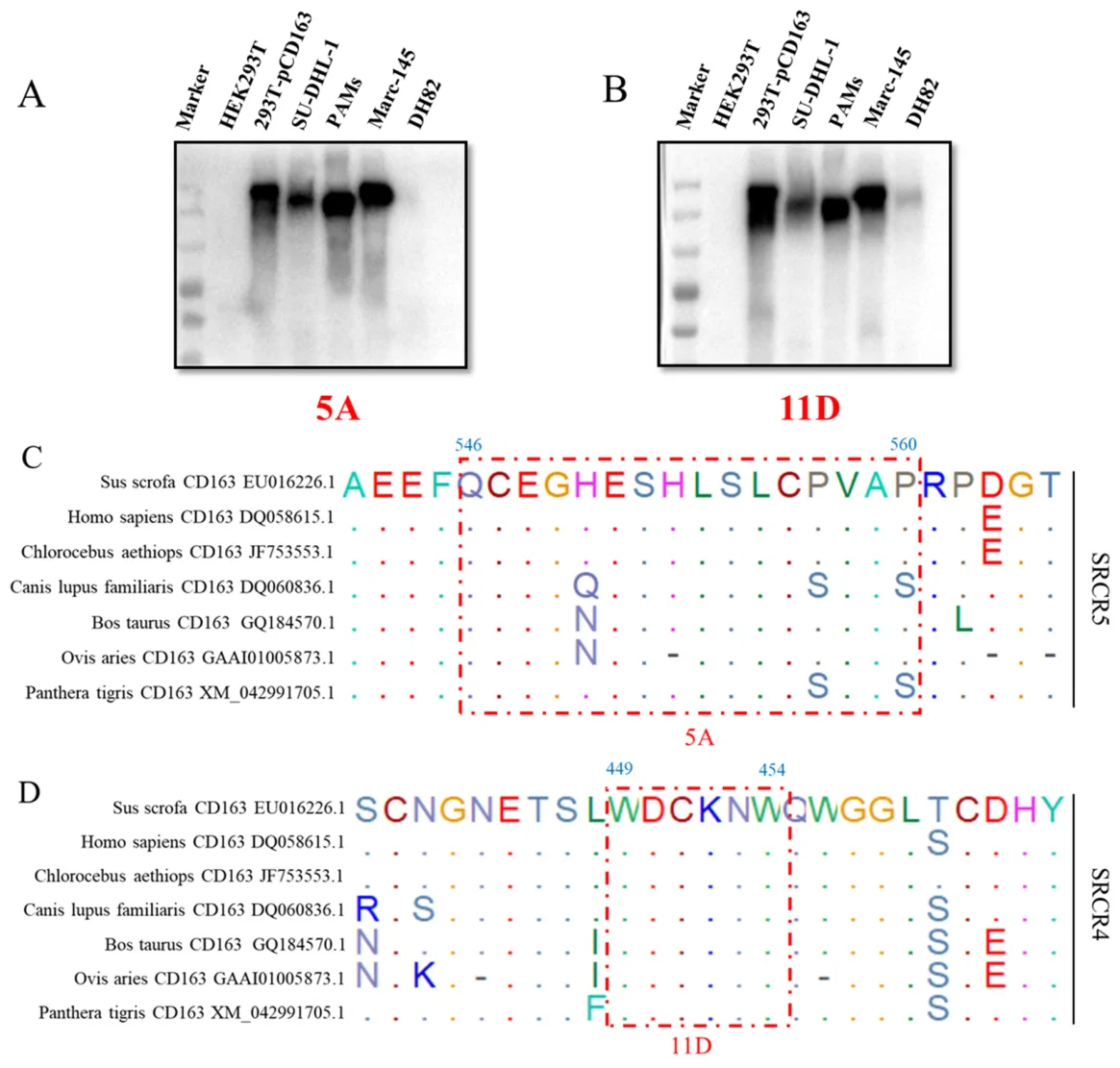
Cross-species reactivity of mAbs 5A and 11D. (**A**,**B**) Western blotting detection of CD163 in SU-DHL-1, PAMs, Marc-145 and DH82 cells by mAbs 5A and 11D, respectively. (**C**,**D**) The alignment of corresponding epitopes recognized by mAbs 5A and 11D in CD163 sequences from different species. The HEK293T cells that did not express CD163 were set as a negative control, while the 293T-pCD163 cells (HEK293T cells stably expressing porcine CD163) were a positive control.

To evaluate the cross-species reactivity of mAbs 5A and 11D, CD163 expression was examined in human (SU-DHL-1), porcine (PAMs), monkey (Marc-145), and canine (DH82) cells. Western blotting showed that mAb 5A recognized CD163 in SU-DHL-1, PAMs, and Marc-145 cells, whereas no reactivity was detected in DH82 cells (Figure 7A). In contrast, mAb 11D recognized CD163 in all four cell types (Figure 7B). Sequence alignment further demonstrated that the ^546^CEGHESHLSLCPVAP^560^ epitope recognized by mAb 5A is conserved in porcine, human and monkey CD163 but not in that of other species (Figure 7C), while the ^449^WDCKNW^454^ epitope recognized by mAb 11D is highly conserved among CD163 sequences from different species (Figure 7D).

## 4. Discussion

Since its emergence in the late 1980s, PRRSV has remained a major economic burden on the global swine industry. A 2025 report showed that PRRSV caused annual losses of 1.2 billion dollars, representing a substantial increase from the 663.91 million dollars reported in 2013 [35]. Vaccine immunization has been the most commonly used strategy to control PRRS in the past 30 years; however, PRRS is still widely spread all around the world [36]. Recent advances in research on PRRSV receptors have opened new avenues for the prevention and control of PRRS. Several studies have confirmed that CD163 is an indispensable receptor for PRRSV entry into target cells [23,37]. CD163-knockout pigs were fully protected from PRRSV infection [16]. Further studies demonstrated that pigs lacking the SRCR5 domain of CD163 are highly resistant to both PRRSV-1 and PRRSV-2 infection [17,19]. In 2025, the FDA approved CD163-SRCR5-knockout pigs [21,22]. Therefore, CD163, especially its SRCR5 domain, has become a Jack-of-all-trades for anti-PRRSV research [38].

Several mAbs against porcine CD163 have been developed [24,25,37]. mAb 2A10 recognizes an epitope in SRCR1-3, which did not affect PRRSV infection but inhibited African swine fever virus infection [37]. mAb 1D7 recognizes an epitope (^497^TWGTVCDSDF^506^) in the SRCR5 domain of porcine CD163, which could partially inhibit HP-PRRSV-2 HuN4-EGFP infection in PAMs [25]. Two additional mAbs (6E8 and 9A10) that recognize the conformational epitopes ^570^SXDVGXV^576^ in SRCR5 and Q^797^ in SRCR7 could inhibit different PRRSV strains via receptor blocking and transcription suppression [24]. In addition, seven nanobodies (Nb1–Nb7) against CD163 SRCR5-9 have been prepared, which displayed strong affinity to the porcine CD163 receptor and broad anti-PRRSV activities (especially Nb2) [26]. In this study, we generated two mAbs (5A and 11D) against porcine CD163, recognizing novel epitopes that have never been identified.

mAb 5A recognizes a new epitope (^546^CEGHESHLSLCPVAP^560^) in SRCR5, located within the long loop 5-6, which is critical for ligand binding [34]. Notably, the epitope is exactly adjacent to the R561 residue, which is critical for PRRSV infection [20]. Therefore, we estimated the anti-PRRSV activity of mAb 5A, showing that it could significantly inhibit some lineage 1 (NADC34-like PRRSV-2) and lineage 8 (HP-PRRSV-2) strains but not the other PRRSV strains. PRRSV has been prevalent in China for 30 years. Currently, lineage 1 and lineage 8 PRRSV isolates are the most prevalent PRRSV in Chinese swine herds [10,28]. This new mAb may not only serve as a potential material for PRRSV treatment but also be used for research of anti-PRRSV mechanisms, especially considering the different antiviral effects of mAb 5A against distinct PRRSV strains. mAb 11D recognizes another new epitope in SRCR4 (^449^WDCKNW^454^), which did not present observable anti-PRRSV effects. These results are consistent with previous studies showing that SRCR5 is essential for PRRSV infection while SRCR4 is dispensable [2,16,19,37].

Besides playing an essential role in PRRSV infection, CD163 is also a dynamic barrier to host-switching of arteriviruses [8,39]. CD163 from apes can effectively support SHFV infection, but CD163 from Old World monkeys and New World monkeys has obviously lower efficiency in supporting SHFV entry. Remarkably, CD163 from humans is fully functional for both SHFV and PRRSV infection, raising a potential spillover threat of arteriviruses to public health [8,39]. However, the exact determining region of CD163 for cross-species infection is still unknown. In this study, mAb 5A, targeting SRCR5, can interact with human, pig and monkey CD163, while mAb 11D, targeting SRCR4, can react with CD163 from a broader range of species, including humans, pigs, monkeys, dogs and so on. Therefore, these mAbs will serve as essential tools for mapping functional CD163 domains, comparing receptor usage among arteriviruses, identifying host-range determinants, and estimating CD163-dependent cross-species potential.

This study still has some limitations. Firstly, the potential antiviral mechanisms of mAb 5A need to be explored. It might act through steric hindrance of GP2a/GP4 binding by inducing conformational changes in CD163 or by altering receptor trafficking. Secondly, the distinct antiviral activities against different PRRSV strains deserve further investigation. They might be due to strain-specific GP2a/GP4 interactions with CD163, differences in receptor usage, or structural differences among viral glycoproteins. Thirdly, the antiviral activity of mAb 5A in vivo needs to be determined in a future study. Fourthly, additional experiments are warranted to validate the practical applicability of these mAbs for CD163-domain-dependent cross-species infection. Fifthly, the antibody isotypes of mAbs 5A and 11D were not determined in this study.

## 5. Conclusions

In this study, we developed two mAbs (5A and 11D) against porcine CD163. mAb 5A recognizes the ^546^CEGHESHLSLCPVAP^560^ epitope in SRCR5, which is located within the long loop 5-6 and is exactly adjacent to the R561 residue of SRCR5, which is critical for PRRSV infection. mAb 11D recognizes the ^449^WDCKNW^454^ epitope in SRCR4, which is highly conserved among different species. Further analysis confirmed that mAb 5A, but not mAb 11D, has an anti-PRRSV effect, while mAb 11D has broader cross-species reactivity than mAb 5A. This study provides essential tools for exploring CD163-domain-dependent cross-species reactivity and anti-PRRSV effects.

## Data Availability

The original contributions presented in this study are included in the article/Supplementary Material. Further inquiries can be directed to the corresponding author.

## Supplementary Materials

The following supporting information can be downloaded at https://www.mdpi.com/article/10.3390/vetsci13080835/s1: Table S1: The primers used in this study. Figure S1: Antiviral effects of mAbs 5A and 11D against different PRRSV strains in Marc-145 cells at 48 hpi. (A,B) The mAb 5A has dose-dependent antiviral activity against NADC34-like rBJ-VVL-P30 strain, but the mAb 11D has not. (C,D) The mAbs 5A and 11D have not antiviral activities against NADC30-like rSD-VVL strain. (E,F) Both mAbs 5A and 11D have not antiviral activities against classical CH-1R strain. File S1: Original images of the maintext.
