# Supplementary material for: Cross-Species Reactivity and Differential Anti-PRRSV Activities of CD163 SRCR4/SRCR5 Monoclonal Antibodies
Source: Vet Sci. 2026 Aug 20;13(8):835. doi: 10.3390/vetsci13080835 (PMC13517797; doi:10.3390/vetsci13080835)

1.A

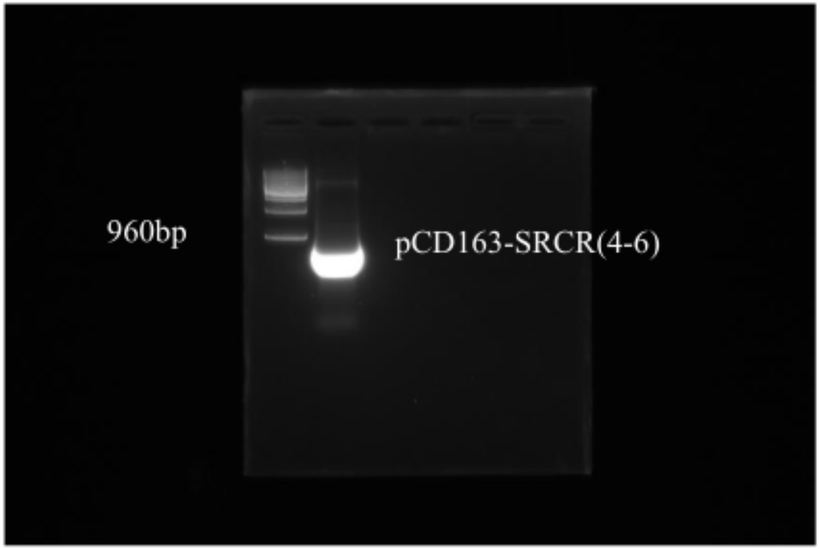

1.B

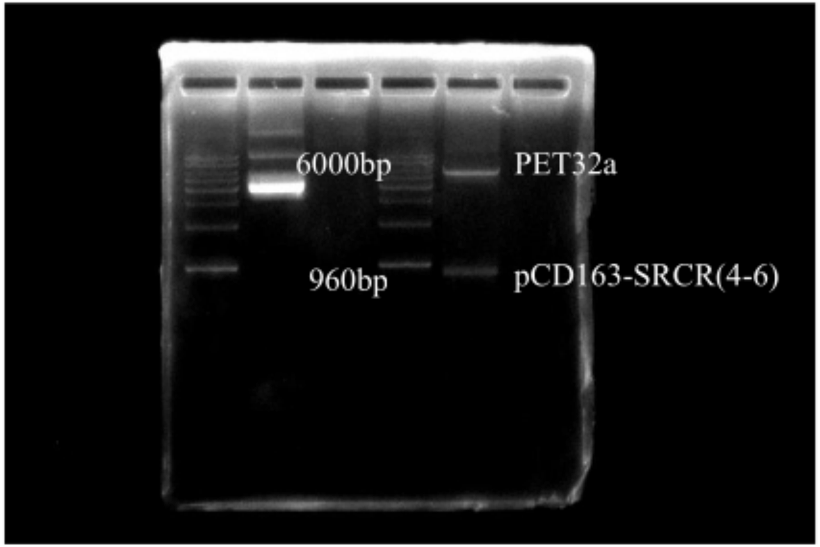

1.C

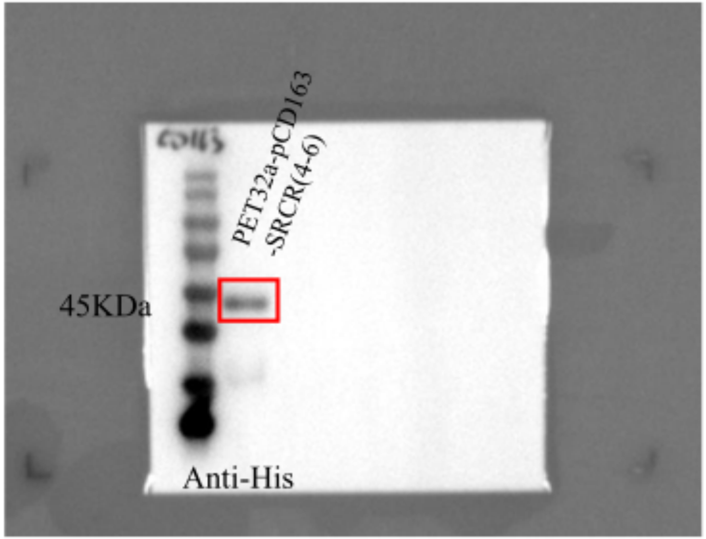

r 1.D

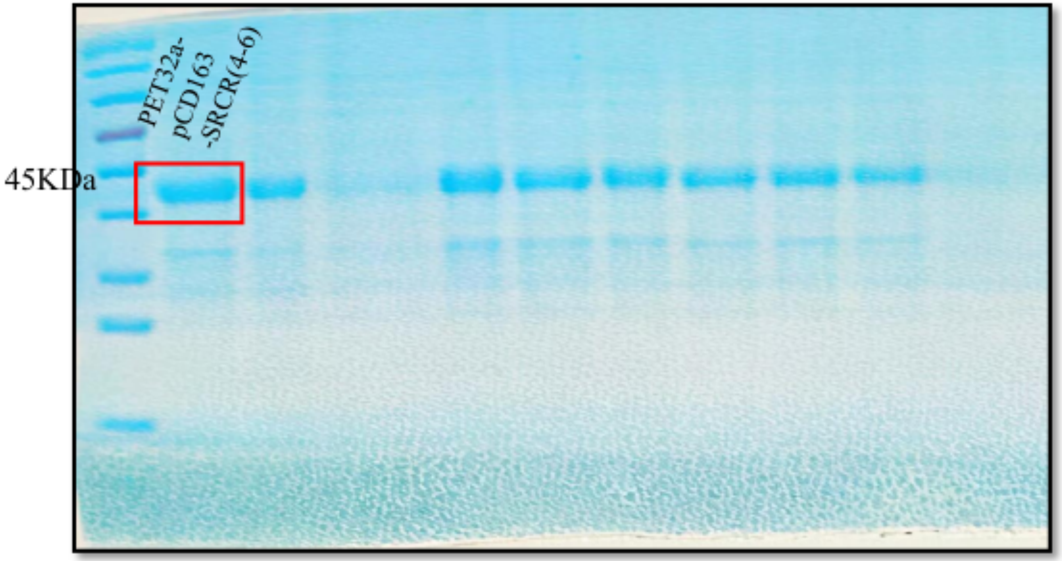

2.A

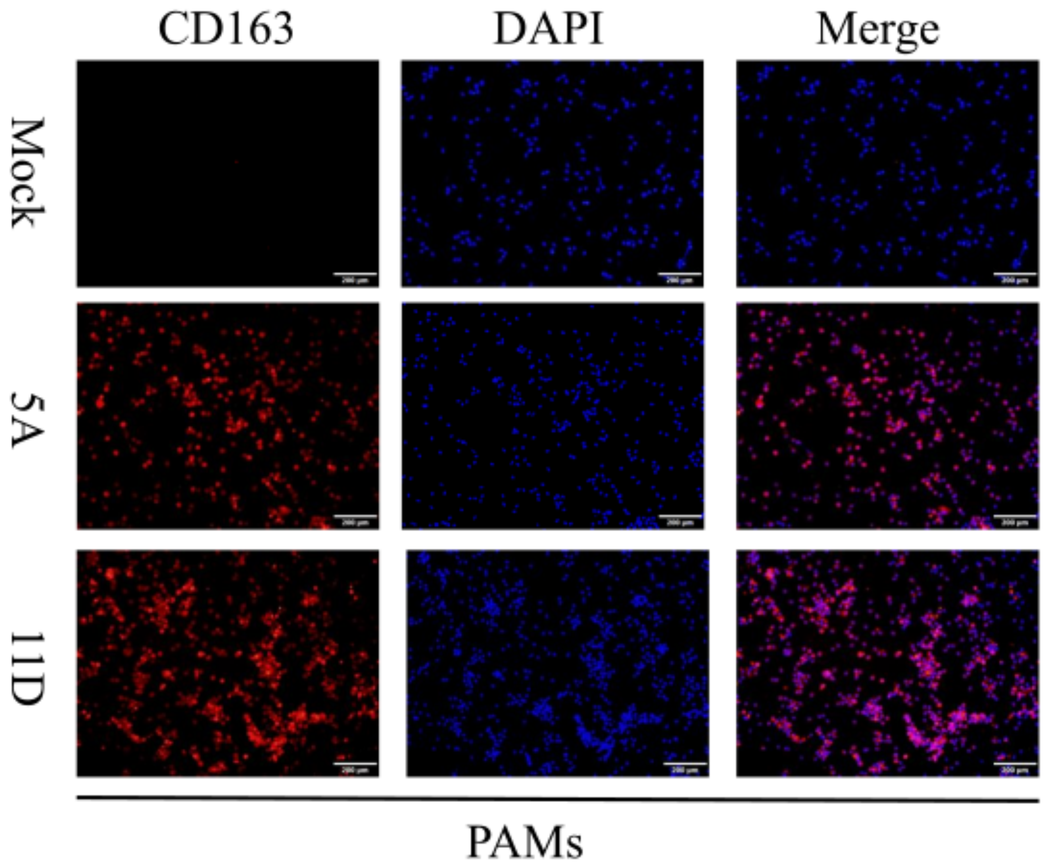

r 3. A

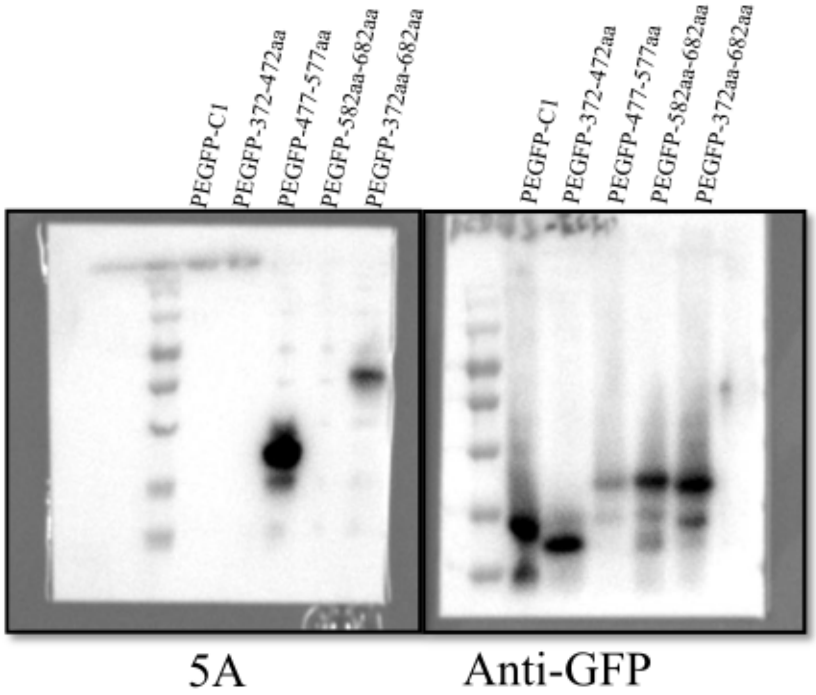
PEGFP-C1
PEGFP-372-472aa
PEGFP-477-577aa
PEGFP-582aa-682aa
PEGFP-372aa-682aa
PEGFP-C1
PEGFP-372-472aa
PEGFP-477-577aa
PEGFP-582aa-682aa
PEGFP-372aa-682aa
5A
Anti-GFP

r 3. B

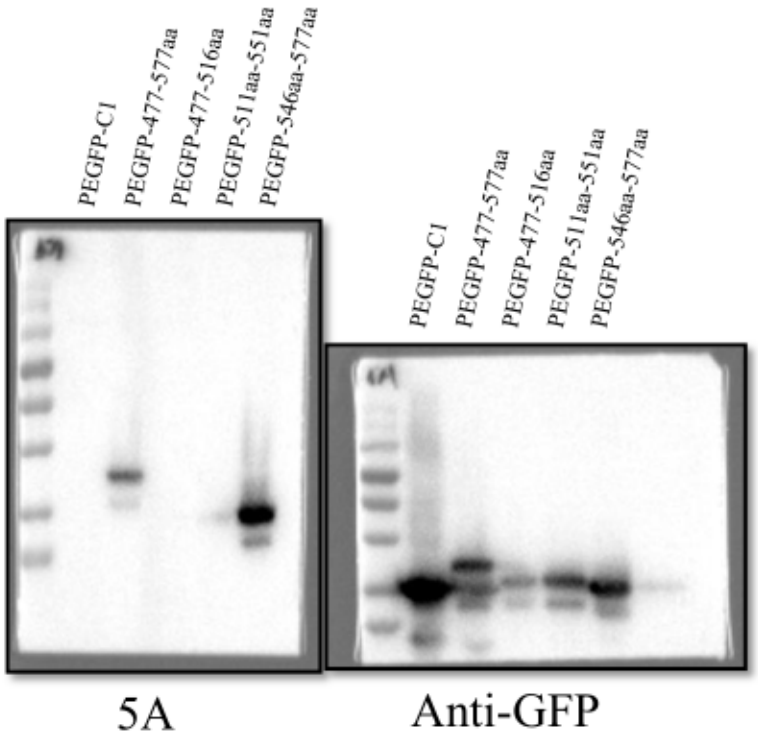
PEGFP-C1
PEGFP-477-577aa
PEGFP-477-516aa
PEGFP-511aa-551aa
PEGFP-546aa-577aa
PEGFP-C1
PEGFP-477-577aa
PEGFP-477-516aa
PEGFP-511aa-551aa
PEGFP-546aa-577aa
5A
Anti-GFP

3. C

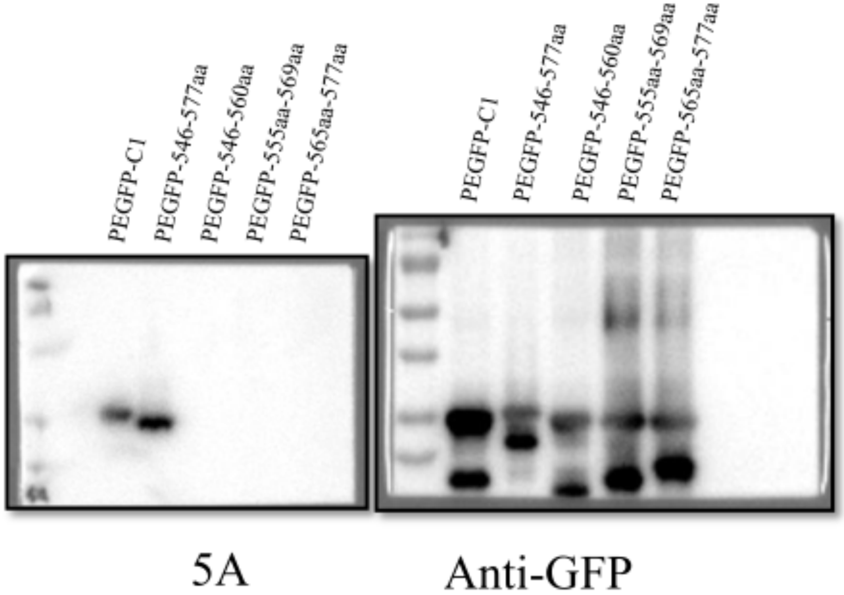

PEGFP-C1
PEGFP-546-577aa
PEGFP-546-560aa
PEGFP-555aa-569aa
PEGFP-565aa-577aa
PEGFP-C1
PEGFP-546-577aa
PEGFP-546-560aa
PEGFP-555aa-569aa
PEGFP-565aa-577aa
5A
Anti-GFP

r 3. D

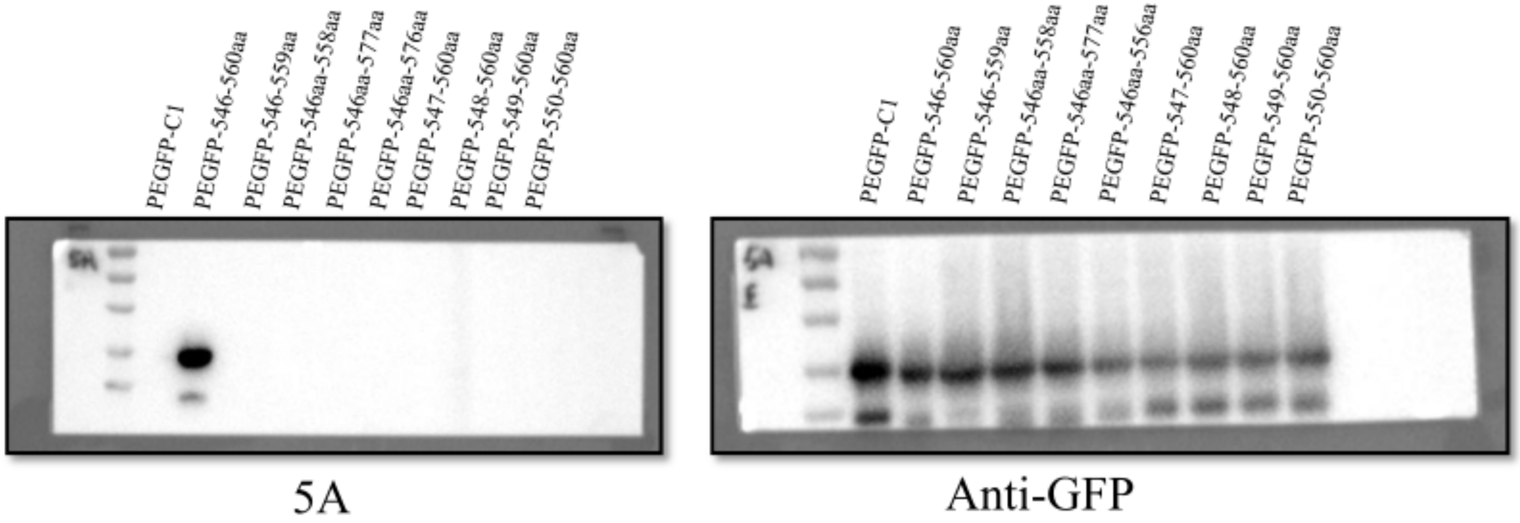
PEGFP-C1
PEGFP-546-560aa
PEGFP-546-559aa
PEGFP-546aa-558aa
PEGFP-546aa-577aa
PEGFP-546aa-576aa
PEGFP-547-560aa
PEGFP-548-560aa
PEGFP-549-560aa
PEGFP-550-560aa
5A
PEGFP-C1
PEGFP-546-560aa
PEGFP-546-559aa
PEGFP-546aa-558aa
PEGFP-546aa-577aa
PEGFP-546aa-556aa
PEGFP-547-560aa
PEGFP-548-560aa
PEGFP-549-560aa
PEGFP-550-560aa
Anti-GFP

4. A

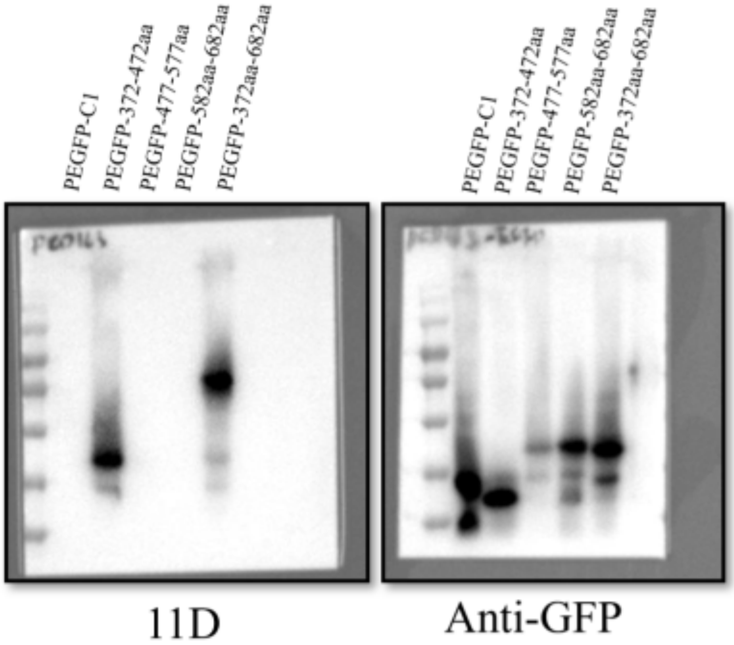
PEGFP-C1
PEGFP-372-472aa
PEGFP-477-577aa
PEGFP-582aa-682aa
PEGFP-372aa-682aa
PEGFP-C1
PEGFP-372-472aa
PEGFP-477-577aa
PEGFP-582aa-682aa
PEGFP-372aa-682aa
11D
Anti-GFP

r 4. B

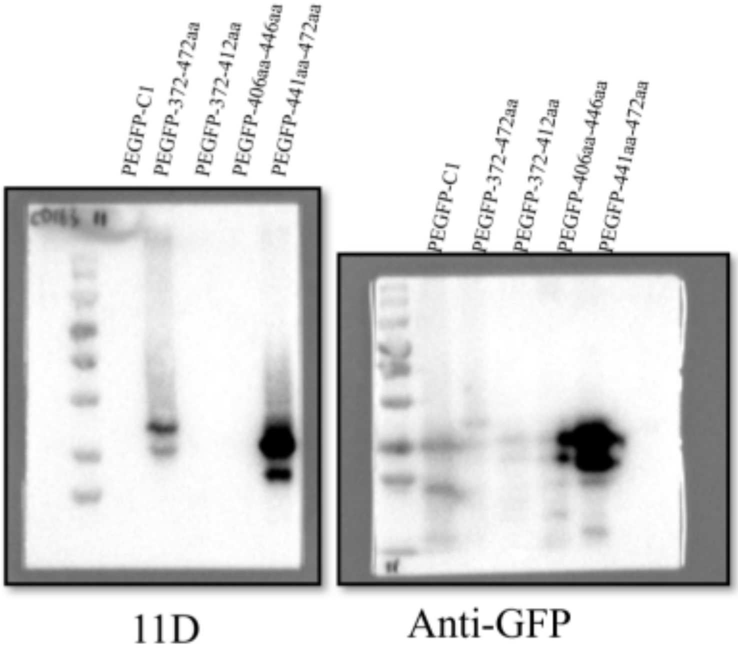
PEGFP-C1
PEGFP-372-472aa
PEGFP-372-412aa
PEGFP-406aa-446aa
PEGFP-441aa-472aa
PEGFP-C1
PEGFP-372-472aa
PEGFP-372-412aa
PEGFP-406aa-446aa
PEGFP-441aa-472aa
11D
Anti-GFP

4. C

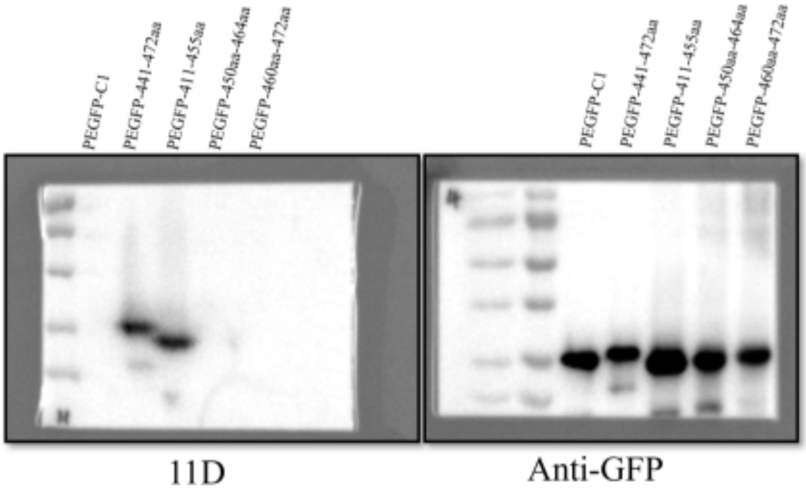
PEGFP-C1
PEGFP-441-472aa
PEGFP-411-455aa
PEGFP-450aa-464aa
PEGFP-460aa-472aa
PEGFP-C1
PEGFP-441-472aa
PEGFP-411-455aa
PEGFP-450aa-464aa
PEGFP-460aa-472aa
11D
Anti-GFP

4. D

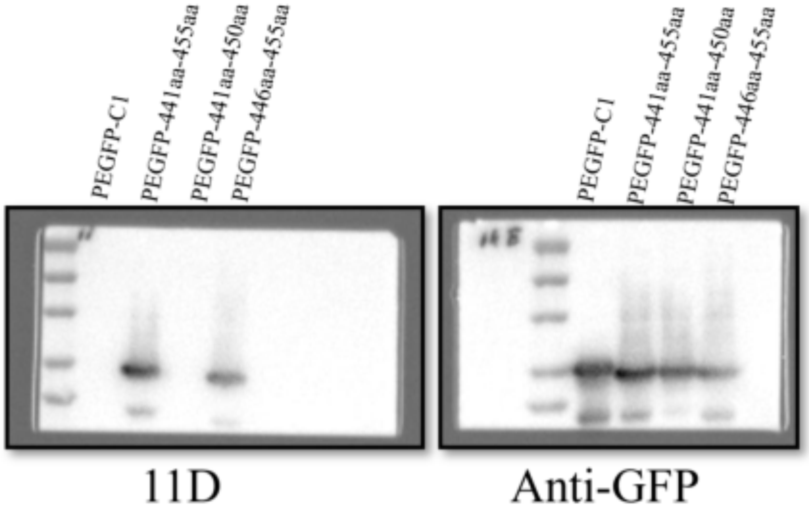
PEGFP-C1
PEGFP-441aa-455aa
PEGFP-441aa-450aa
PEGFP-446aa-455aa
11D
PEGFP-C1
PEGFP-441aa-455aa
PEGFP-441aa-450aa
PEGFP-446aa-455aa
Anti-GFP

4. E

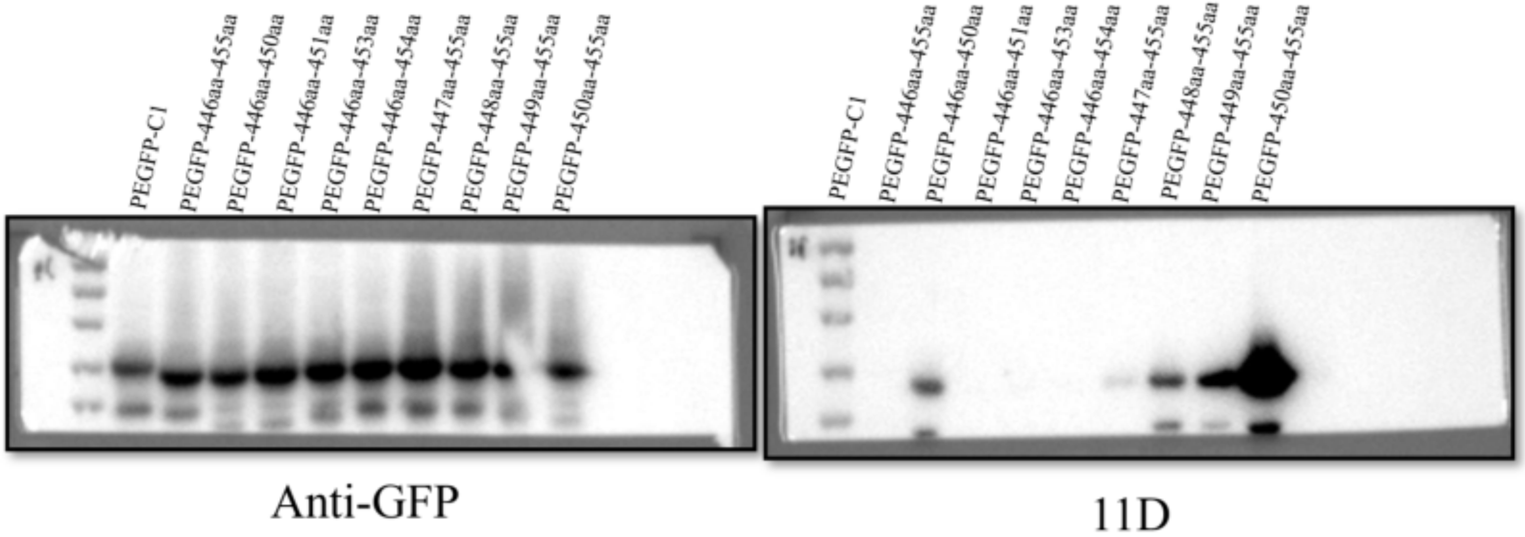
PEGFP-C1
PEGFP-446aa-455aa
PEGFP-446aa-450aa
PEGFP-446aa-451aa
PEGFP-446aa-453aa
PEGFP-446aa-454aa
PEGFP-447aa-455aa
PEGFP-448aa-455aa
PEGFP-449aa-455aa
PEGFP-450aa-455aa
Anti-GFP
PEGFP-C1
PEGFP-446aa-455aa
PEGFP-446aa-450aa
PEGFP-446aa-451aa
PEGFP-446aa-453aa
PEGFP-446aa-454aa
PEGFP-447aa-455aa
PEGFP-448aa-455aa
PEGFP-449aa-455aa
PEGFP-450aa-455aa
11D

r 6.A-B

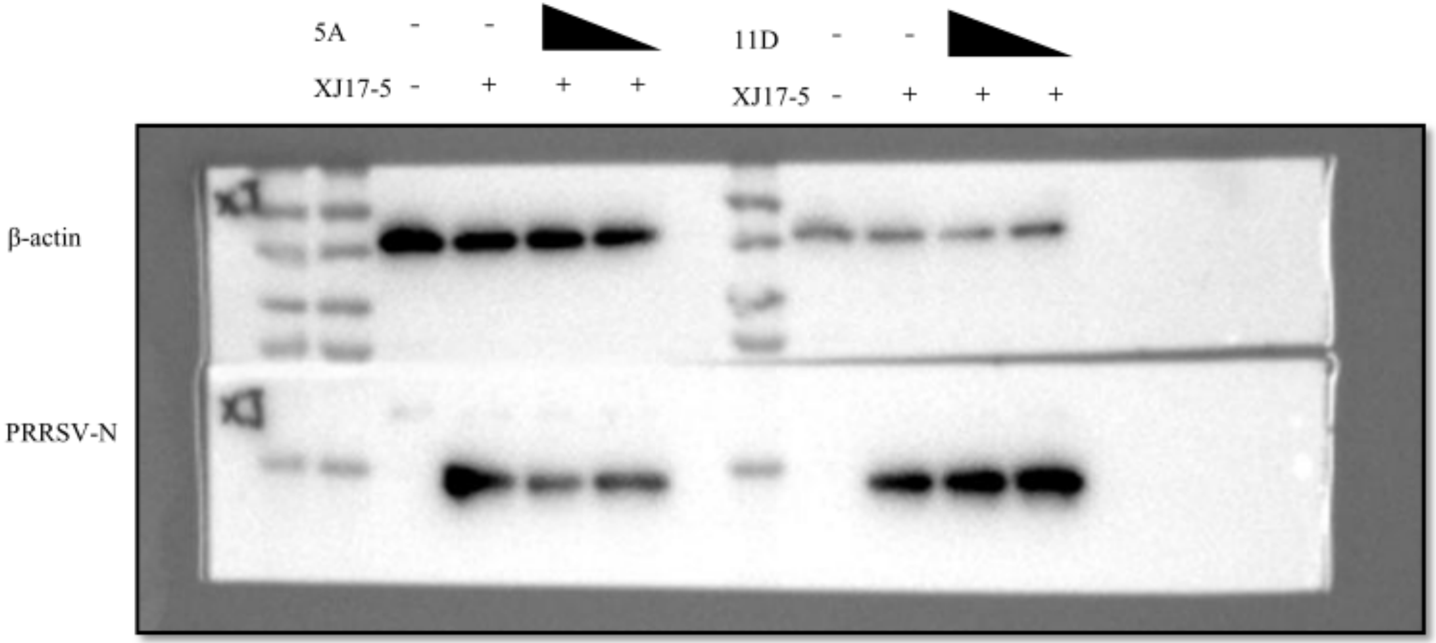

r 6.C

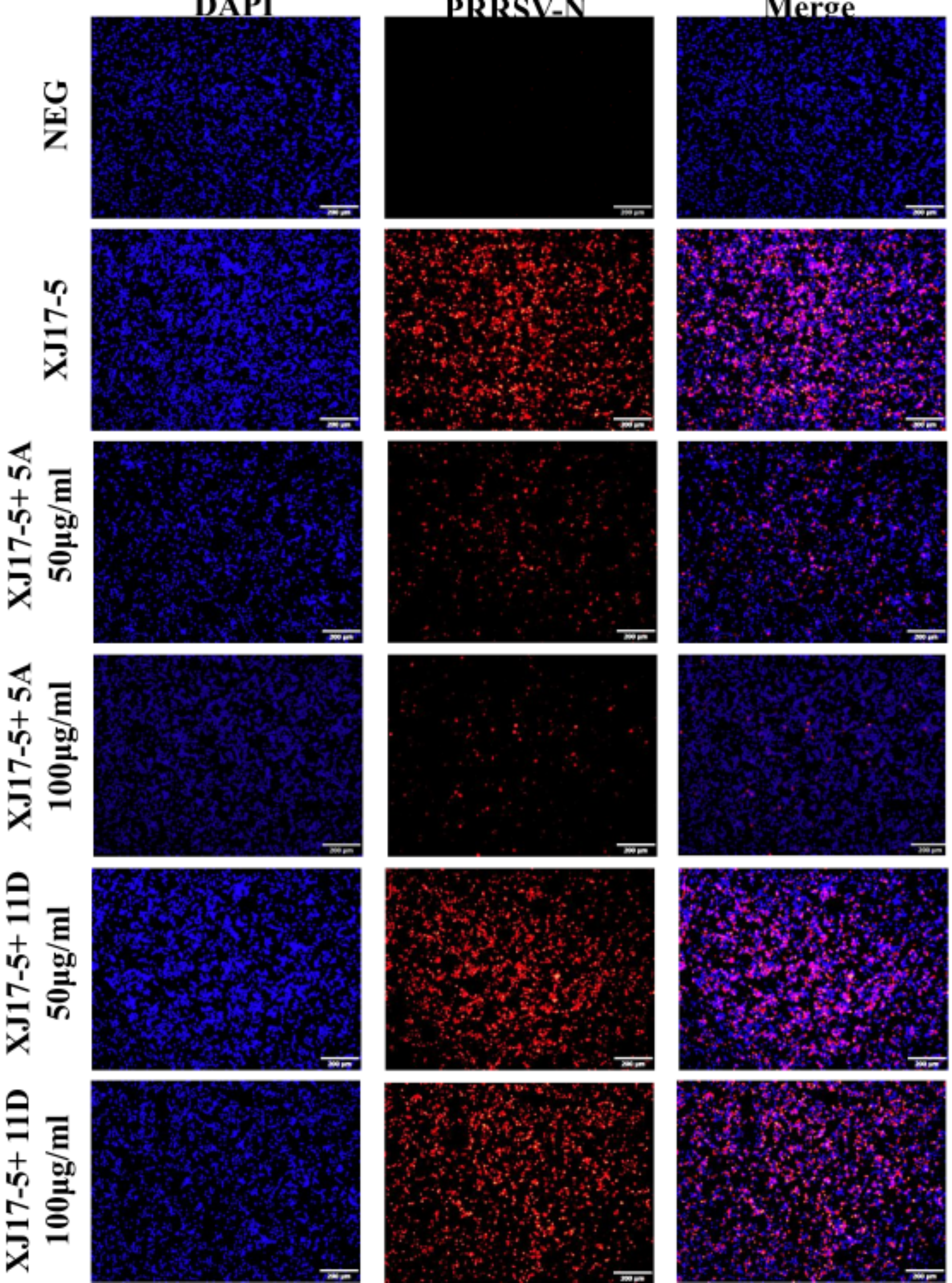
DAPI
PRRSV-N
Merge
NEG
XJ17-5
XJ17-5+ 5A 50μg/ml
XJ17-5+ 5A 100μg/ml
XJ17-5+ 11D 50μg/ml
XJ17-5+ 11D 100μg/ml

7.A

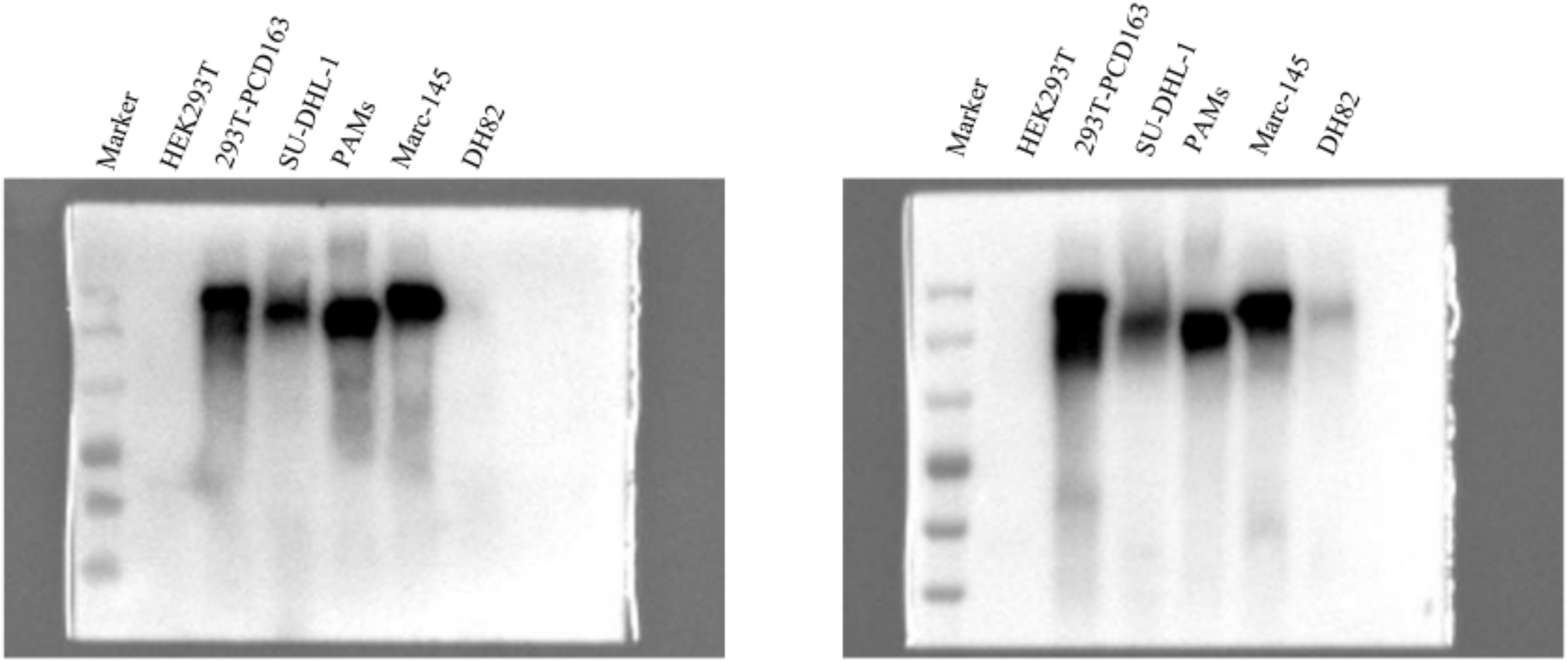
Marker
HEK293T
293T-PCD163
SU-DHL-1
PAMs
Marc-145
DH82
Marker
HEK293T
293T-PCD163
SU-DHL-1
PAMs
Marc-145
DH82

S1.A-B

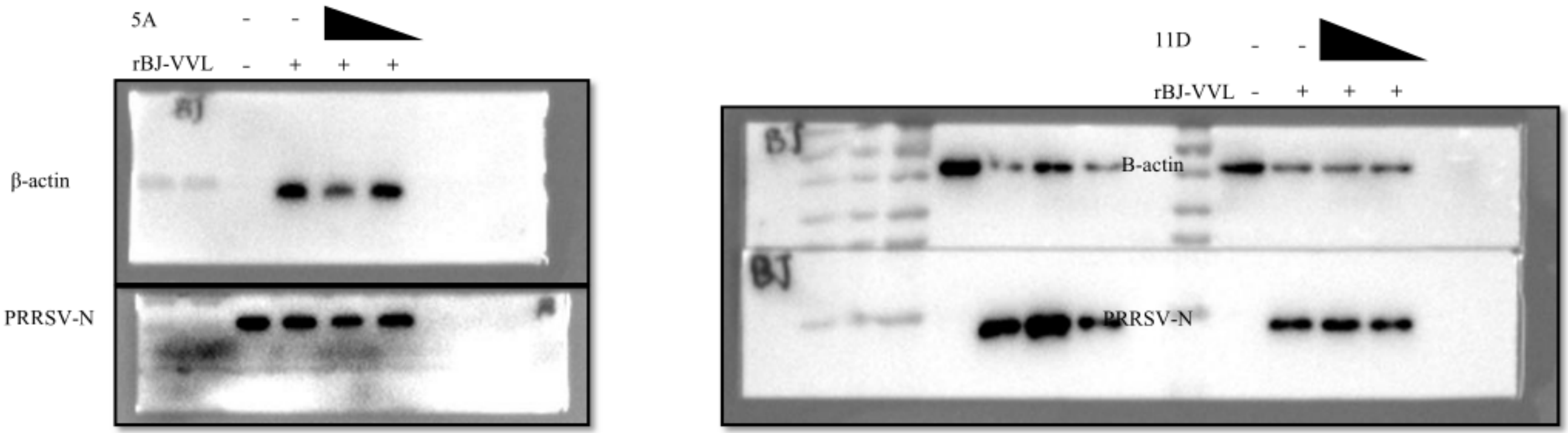

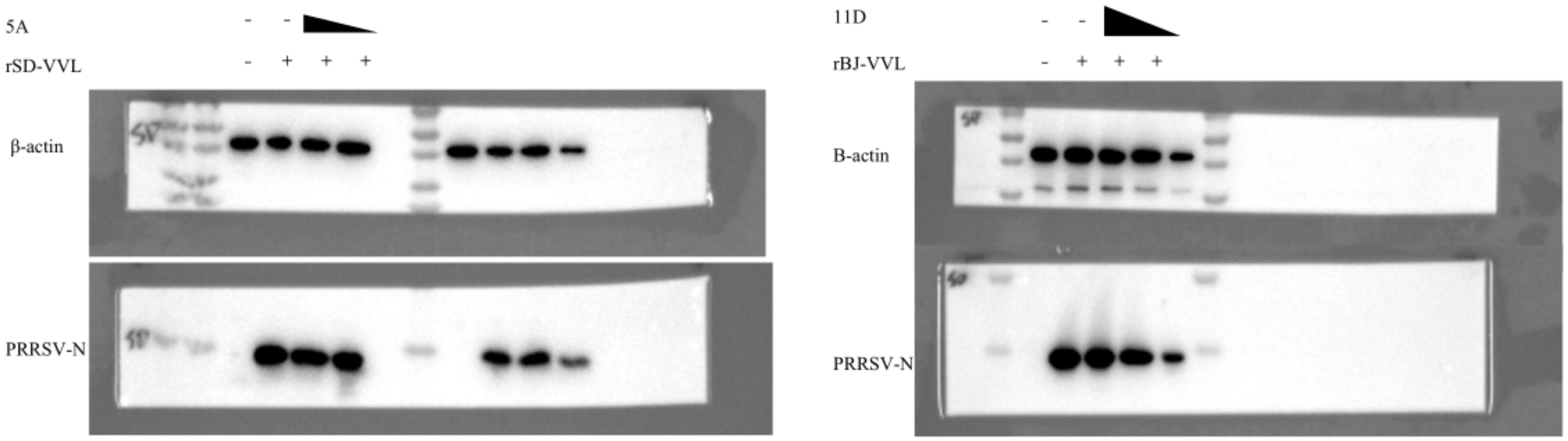

S1. C-D

s1.E-F

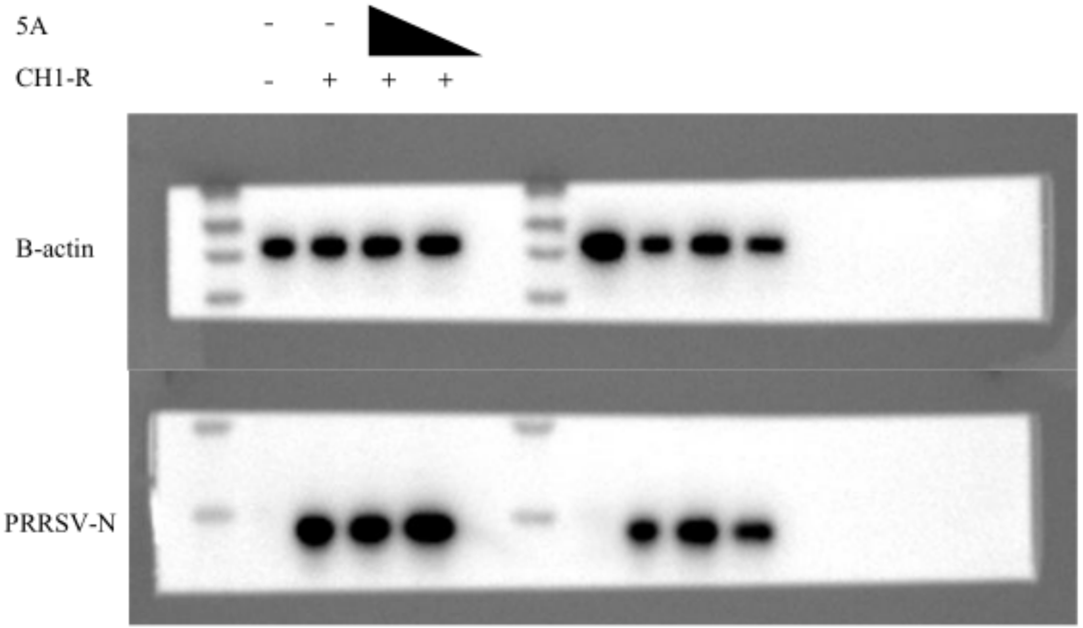

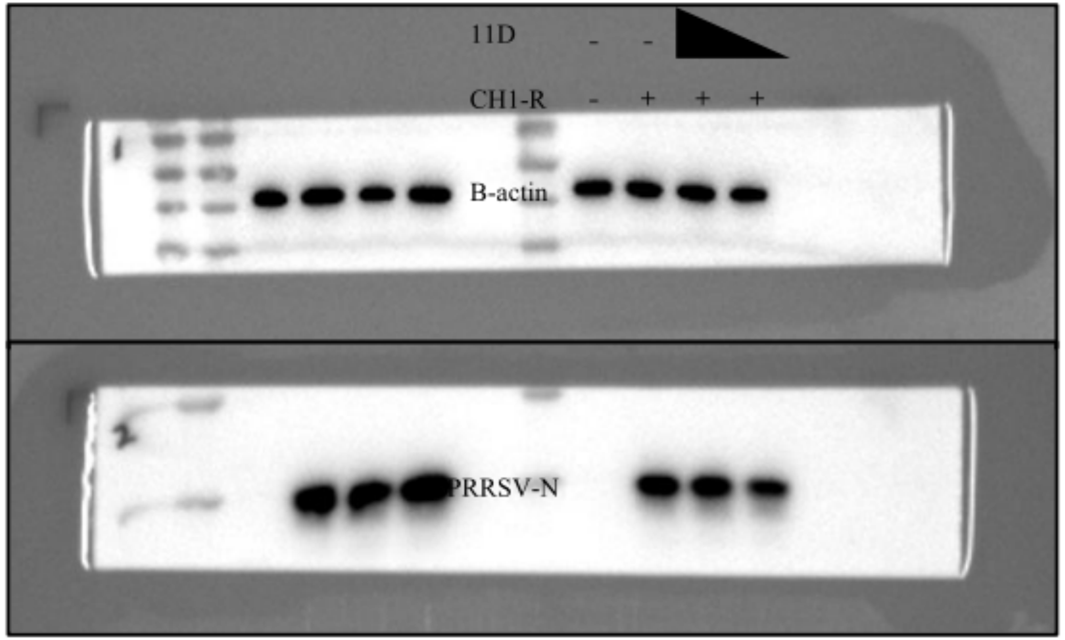

Supplement: Supplementary file 1 [file vetsci-13-00835-s001.zip › vetsci-4479808-File S1-Original images of the maintext.pdf]
