# Supplementary material for: Cross-Species Reactivity and Differential Anti-PRRSV Activities of CD163 SRCR4/SRCR5 Monoclonal Antibodies
Source: Vet Sci. 2026 Aug 20;13(8):835. doi: 10.3390/vetsci13080835 (PMC13517797; doi:10.3390/vetsci13080835)
Supplement: Supplementary file 1 [file vetsci-13-00835-s001.zip › vetsci-4479808-Table S1 and Figure S1.pdf]

Table S1. The primers used in this study.

No.	Name	Sequence（5′-3′）
1	pET32a-N-6his-F	ATGCACCATCATCATCATCATTCTTCTGGTCTGGTGCCACGCG
2	pET32a-N-6his-R	AGTGGTGGTGGTGGTGGTGCTCGAGTGAGCAGATTACAGAGGCCACTTGCC
3	SRCR4-F	GCTGTACAAGTCCGGACTCAGATCTCTGAGACTTAAAGGTGGAGGCAGCC
4	SRCR4-R	TGGATCCCGGGCCCGCGGTACCTTATCTCATGAGCAGGTAATTTTGGCTTCG
5	SRCR5-F	GACGAGCTGTACAAGTCCGGACTCAGATCTGCCCACAGGAAACCCAGGCTGGTT
6	SRCR5-R	GATCCCGGGCCCGCGGTACCTTATTGTGTGTATCTTGAGCAGACTACGCCGAC
7	SRCR6-F	CTGTACAAGTCCGGACTCAGATCTATCCGCTTGGTGAATGGCAAG
8	SRCR6-R	TGGTGGTGGTGGTGCTCGAGTGAGCAGATTACAGAGGCCAC
9	5-1-40-R	GATCCCGGGCCCGCGGTACCTTACCTGCACAGCACGCT
10	5-35-75-F	GACGAGCTGTACAAGTCCGGACTCAGATCTGCCAGCGTGCTGTGCAGGGAACTACAGT
11	5-35-75-R	GATCCCGGGCCCGCGGTACCTTAGGACTCGTGCCCCTCACACTGGAATTC
12	5-75-105-F	GACGAGCTGTACAAGTCCGGACTCAGATCTTGTGAGGGGCACGAGTCCCACCTTT
13	5-70-85-R	GATCCCGGGCCCGCGGTACCTTAGGGTGCTACTGGGCAGAGTG
14	5-80-95-F	GACGAGCTGTACAAGTCCGGACTCAGATCTCTCTGCCCAGTAGCA
15	5-80-95-R	GATCCCGGGCCCGCGGTACCTTAGCTGTGGCTACATGTCCC
16	5-90-101-F	GACGAGCTGTACAAGTCCGGACTCAGATCTACATGTAGCCACAGCAGGGAC
17	5-90-101-R	GATCCCGGGCCCGCGGTACCTTATTGTGTGTATCTTGAGCAGAC
18	5-75-85-F	GCTGTACAAGTCCGGACTCAGATCTACTTCTCTTTGGGACTGCAAGAATTGGCAG
19	5-75-85-R	TGGATCCCGGGCCCGCGGTACCTTACTGCCAATTCTTGCAGTCCCAAAGAGAAGT
20	5-70-84-F1-F	TGTGAGGGGCACGAGTCCCACCTTTCACTCTGCCCAGTAGCATAAGGTACCGCGGGCCCGGGAT
21	5-70-84-F1-R	GGACCGAACCCCGCGTTTATGAAC
22	5-70-84-F2-F	GTTCATAAACGCGGGGTTCGGTCC
23	5-70-84-F2-R	TGCTACTGGGCAGAGTGAAAGGTGGGACTCGTGCCCCTCACAAGATCTGAGTCCGGACTTGTAC
24	5-70-83-F1-F	TGTGAGGGGCACGAGTCCCACCTTTCACTCTGCCCAGTATAAGGTACCGCGGGCCCGGGATC
25	5-70-83-F2-R	TACTGGGCAGAGTGAAAGGTGGGACTCGTGCCCCTCACAAGATCTGAGTCCGGACTTGTACAG
26	5-70-82-F1-F	TGTGAGGGGCACGAGTCCCACCTTTCACTCTGCCCATAAGGTACCGCGGGCCCGGGATC
27	5-70-82-F2-R	TGGGCAGAGTGAAAGGTGGGACTCGTGCCCCTCACAAGATCTGAGTCCGGACTTGTACAGC
28	5-70-81-F1-F	TGTGAGGGGCACGAGTCCCACCTTTCACTCTGCTAAGGTACCGCGGGCCCGGGATC
29	5-70-81-F2-R	GCAGAGTGAAAGGTGGGACTCGTGCCCCTCACAAGATCTGAGTCCGGACTTGTACAGC
30	5-71-85-F1-F	GAGGGGCACGAGTCCCACCTTTCACTCTGCCCAGTAGCACCCTAAGGTACCGCGGGCCCGGGAT
31	5-71-85-F2-F	GGGTGCTACTGGGCAGAGTGAAAGGTGGGACTCGTGCCCCTCAGATCTGAGTCCGGACTTGTA
32	5-72-85-F1-F	GGGCACGAGTCCCACCTTTCACTCTGCCCAGTAGCACCCTAAGGTACCGCGGGCCCGGGAT
33	5-72-85-F2-F	GGGTGCTACTGGGCAGAGTGAAAGGTGGGACTCGTGCCCAGATCTGAGTCCGGACTTGTAC
34	5-73-85-F1-F	CACGAGTCCCACCTTTCACTCTGCCCAGTAGCACCCTAAGGTACCGCGGGCCCGGGAT
35	5-73-85-F2-F	GGGTGCTACTGGGCAGAGTGAAAGGTGGGACTCGTGAGATCTGAGTCCGGACTTGTAC
36	5-74-85-F1-F	GAGTCCCACCTTTCACTCTGCCCAGTAGCACCCTAAGGTACCGCGGGCCCGGGAT
37	5-74-85-F2-F	GGGTGCTACTGGGCAGAGTGAAAGGTGGGACTCAGATCTGAGTCCGGACTTGTAC
38	4-1-40-R	TGGATCCCGGGCCCGCGGTACCTTATCTCACCTGCAAACCACATCAG
39	4-35-75-F	GCTGTACAAGTCCGGACTCAGATCTGCTGATGTGGTTTGCAGGCAG
40	4-35-75-R	TGGATCCCGGGCCCGCGGTACCTTATCTCAAGTTTCATTTCCATTACAGCTGC
41	4-70-101-F	GCTGTACAAGTCCGGACTCAGATCTTGTAATGGAAATGAAACTTCTCTTTGGG

42	4-70-85AA-F	GCTGTACAAGTCCGGACTCAGATCTTGTAATGGAAATGAAACTTC
43	4-75-85AA-R	TGGATCCCGGGCCCGCGGTACCTTACTGCCAATTCTTGCAGTCCC
44	4-80-95AA-F	GCTGTACAAGTCCGGACTCAGATCTGACTGCAAGAATTGGCAGTGG
45	4-80-95AA-R	TGGATCCCGGGCCCGCGGTACCTTAATAGTGATCACAACTAAGTCC
46	4-90-101AA-F	GCTGTACAAGTCCGGACTCAGATCTAGTTGTGATCACTATGACGA
47	4-90-101-AA-R	TGGATCCCGGGCCCGCGGTACCTTATCTCATGAGCAGGTAATTTTGG
48	4-70-80AA-F	GCTGTACAAGTCCGGACTCAGATCTTGTAATGGAAATGAAACTTCTCTTTGGGAC
49	4-70-80AA-R	TGGATCCCGGGCCCGCGGTACCTTAGTCCCAAAGAGAAGTTTCATTTCCATTACA
50	4-75-85AA-F	GCTGTACAAGTCCGGACTCAGATCTACTTCTCTTTGGGACTGCAAGAATTGGCAG
51	4-75-85AA-R	TGGATCCCGGGCCCGCGGTACCTTACTGCCAATTCTTGCAGTCCCAAAGAGAAGT
52	4-75-84-F1-F	ACTTCTCTTTGGGACTGCAAGAATTGGTAAGGTACCGCGGGCCCGGGATCC
53	4-F1-R	GGACCGAACCCCGCGTTTATGAAC
54	4-75-84-F2-F	CCAATTCTTGCAGTCCCAAAGAGAAGTAGATCTGAGTCCGGACTTGTACAGC
55	4-F2-R	GTTCATAAACGCGGGGTTCGGTCC
56	4-75-83-F1-F	ACTTCTCTTTGGGACTGCAAGAATTAAGGTACCGCGGGCCCGGGATCC
57	4-75-83-F2-F	ATTCTTGCAGTCCCAAAGAGAAGTAGATCTGAGTCCGGACTTGTACAGC
58	4-75-82-F1-F	ACTTCTCTTTGGGACTGCAAGTAAGGTACCGCGGGCCCGGGATCC
59	4-75-82-F2-F	CTTGCAGTCCCAAAGAGAAGTAGATCTGAGTCCGGACTTGTACAG
60	4-75-81-F1-F	ACTTCTCTTTGGGACTGCTAAGGTACCGCGGGCCCGGGATCC
61	4-75-81-F2-F	GCAGTCCCAAAGAGAAGTAGATCTGAGTCCGGACTTGTACAG
62	4-75-80-F1-F	ACTTCTCTTTGGGACTAAGGTACCGCGGGCCCGGGAT
63	4-75-80-F2-F	GTCCCAAAGAGAAGTAGATCTGAGTCCGGACTTGTACAG
64	4-76-85-F1-F	TCTCTTTGGGACTGCAAGAATTGGCAGTAAGGTACCGCGGGCCCGGGAT
65	4-76-85-F2-F	CTGCCAATTCTTGCAGTCCCAAAGAGAAGATCTGAGTCCGGACTTGTACAGC
66	4-77-85-F1-F	CTTTGGGACTGCAAGAATTGGCAGTAAGGTACCGCGGGCCCGGGAT
67	4-77-85-F2-F	CTGCCAATTCTTGCAGTCCCAAAGAGATCTGAGTCCGGACTTGTACAGC
68	4-78-85-F1-F	TGGGACTGCAAGAATTGGCAGTAAGGTACCGCGGGCCCGGGAT
69	4-78-85-F2-F	CTGCCAATTCTTGCAGTCCCAAGATCTGAGTCCGGACTTGTACAGC
70	4-79-85-F1-F	GACTGCAAGAATTGGCAGTAAGGTACCGCGGGCCCGGGAT
71	4-79-85-F2-F	CTGCCAATTCTTGCAGTCAGATCTGAGTCCGGACTTGTACAGC

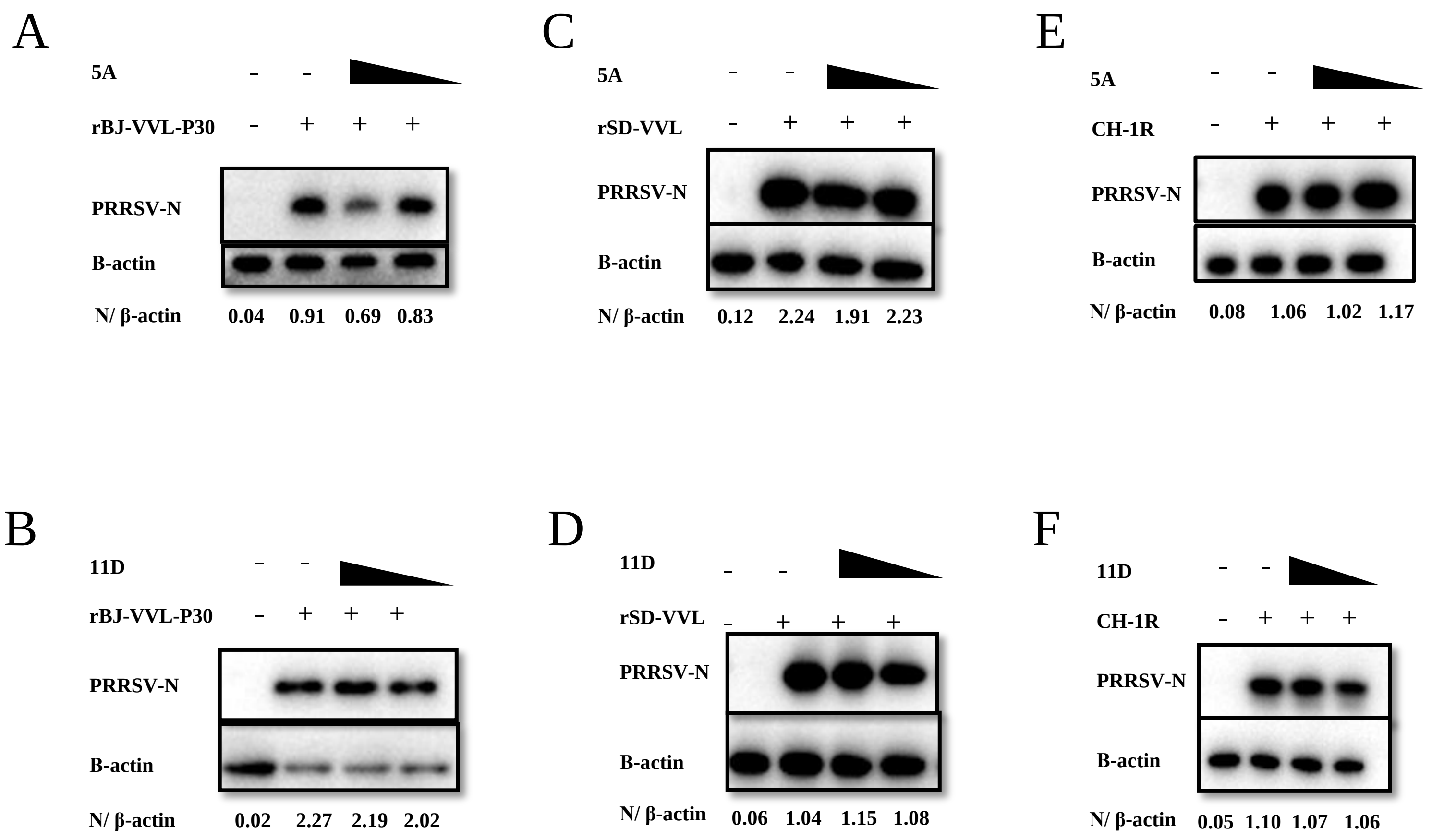

Figure S1. Antiviral effects of mAbs 5A and 11D against different PRRSV strains in Marc-145 cells at 48 hpi. (A-B) The mAb 5A has dose-dependent antiviral activity against NADC34-like rBJ-VVL-P30 strain, but the mAb 11D has not. (C-D) The mAbs 5A and 11D have not antiviral activities against NADC30-like rSD-VVL strain. (E-F) Both mAbs 5A and 11D have not antiviral activities against classical CH-1R strain.
